# Supplementary material for: Epigenome-wide association study of asthma and wheeze in childhood and adolescence
Source: Clin Epigenetics. 2017 Oct 13;9:112. doi: 10.1186/s13148-017-0414-7 (PMC5640901; doi:10.1186/s13148-017-0414-7)
Supplement: Supplementary file 1 — Supplementary Tables and Figures. (DOCX 2448 kb) [file 13148_2017_414_MOESM1_ESM.docx]

**Additional file 1**

**ALSPAC data**

The Avon Longitudinal Study of Parents and Children (ALSPAC) recruited 14,541 pregnant women resident in the former county of Avon, UK with expected dates of delivery 1st April 1991 to 31st December 1992. The initial number of pregnancies was 14,541, for which the mother enrolled in the ALSPAC study and had either returned at least one questionnaire or attended a “Children in Focus” clinic by 19/07/99. Of these initial pregnancies, there was a total of 14,676 fetuses, resulting in 14,062 live births and 13,988 children who were alive at 1 year of age.

When the oldest children were approximately 7 years of age, an attempt was made to bolster the initial sample with cases who had failed to join the study originally. The number of new pregnancies not in the initial sample was 706, resulting in an additional 713 children being enrolled. As a result, when considering variables collected from the age of seven onwards (and potentially abstracted from obstetric notes) there are data available for more than the 14,541 pregnancies mentioned above. The total sample size for analyses using any data collected after the age of seven is therefore 15,247 pregnancies, of these, 14,775 were live births and 14,701 were alive at 1 year of age. The phases of enrolment are described in detail in the cohort profile paper [1].

**DNA methylation data**

Peripheral bloods (whole blood or buffy coats) were collected according to standard procedures, spun and frozen at -80˚C. DNA methylation analysis and data pre-processing were performed at the University of Bristol as part of the ARIES project (ariesepigenomics.org.uk). Following extraction, DNA was bisulfite converted using the Zymo EZ DNA MethylationTM kit (Zymo, Irvine, CA). Following conversion, the genome-wide methylation status of over 485,000 CpG sites was measured using the Illumina Infinium® HumanMethylation450k BeadChip assay according to the standard protocol. The arrays were scanned using an Illumina iScan and initial quality review was assessed using GenomeStudio (version 2011.1). The level of methylation is expressed as a “Beta” value (β-value), ranging from 0 (no cytosine methylation) to 1 (complete cytosine methylation). Samples from all timepoints in ARIES were distributed across slides using a semi-random approach (sampling criteria were in place to ensure that all time-points were represented on each array) to minimize the possibility of confounding by batch effects. In addition, during the data generation process a wide range of batch variables were recorded in a purpose-built laboratory information management system (LIMS). The LIMS also reported QC metrics from the standard control probes on the HumanMethylation450k BeadChip for each sample back to the laboratory. Samples failing quality control (average probe detection p-value ≥ 0.01) were repeated. As an additional quality control step genotype probes on the HumanMethylation450k were compared between samples from the same individual and against SNP-chip data to identify and remove any sample mismatches. Data were pre-processed in R (version 3.0.1) with the WateRmelon package[2] according to the subset quantile normalization approach described by Touleimat & Tost[3] in an attempt to reduce the nonbiological differences between probes.

Sites on sex chromosomes were excluded to reduce complexity due to sex-specific differences and X-chromosome inactivation by DNA methylation in females. Probes identified by Naeem et al.[4] that map to multiple genomic locations, contain known repeat regions, INDELs or SNPs, or are affected by other unknown factors were excluded. Outliers were also removed from the methylation data using Tukey’s method[5] of outlier removal as certain extreme methylation values caused by technical artefacts or rare genetic variants skewed the analysis. Potential outliers in the methylation data were removed if their value was less than the lower quartile minus three times the interquartile range or more than the upper quartile plus three times the interquartile range. Finally, probes showing a detection P-value >0.05 for >5% samples were excluded. This left 285,929 and 285,656 probes for analysis in peripheral blood in childhood and peripheral blood in adolescence, respectively.

Table S 1 Cell counts estimated from methylation at 7.5 years and from methylation at 16.5 years for all ARIES individuals.

|  | **CD8 T** | **CD4 T** | **NK cells** | **B-cells** | **Monocytes** | **Eosinophils** | **Neutrophils** |
| --- | --- | --- | --- | --- | --- | --- | --- |
| **Child (7.5 years)** | | | | | | | |
| Min. | 0.03162 | 0.0253 | 0 | 0.04502 | 0.01579 | 0 | 0.002314 |
| 1^st^ Q | 0.11927 | 0.1455 | 0 | 0.1182 | 0.06561 | 0 | 0.361202 |
| Median | 0.14939 | 0.1797 | 0.00154 | 0.14017 | 0.08016 | 0 | 0.440797 |
| Mean | 0.15053 | 0.181 | 0.01461 | 0.14172 | 0.08123 | 0.02505 | 0.434991 |
| 3^rd^ Q | 0.17937 | 0.2136 | 0.02485 | 0.16391 | 0.09455 | 0.03769 | 0.507454 |
| Max. | 0.28008 | 0.4013 | 0.11079 | 0.25798 | 0.18855 | 0.26875 | 0.789483 |
| **Adolescent (16.5 years)** | | | | | | | |
| Min. | 0 | 0 | 0 | 0 | 0.02135 | 0 | 0.1546 |
| 1^st^ Q | 0.12 | 0.1154 | 0 | 0.08257 | 0.06764 | 0 | 0.4088 |
| Median | 0.1563 | 0.1496 | 0.02132 | 0.10543 | 0.08529 | 0 | 0.4801 |
| Mean | 0.1583 | 0.1509 | 0.03192 | 0.10681 | 0.08619 | 0.005257 | 0.4876 |
| 3^rd^ Q | 0.1904 | 0.1832 | 0.04837 | 0.12931 | 0.10518 | 0 | 0.5587 |
| Max. | 0.5062 | 0.358 | 0.26158 | 0.21345 | 0.20034 | 0.206391 | 0.8825 |

Table S 2 Covariates included in all EWAS models.

| **Covariate** | **Coded as** |
| --- | --- |
| Maternal education | Categorical: University, A level, O level or lower |
| Maternal smoking | Categorical: Never, Temporary, Throughout pregnancy |
| Parity | Categorical: Nulliparous, previous pregnancies |
| Maternal age | Age in months |
| Sex | Male/Female |

Figure S 1 Concept of bi-directional two-sample Mendelian randomization of asthma and DNA methylation.


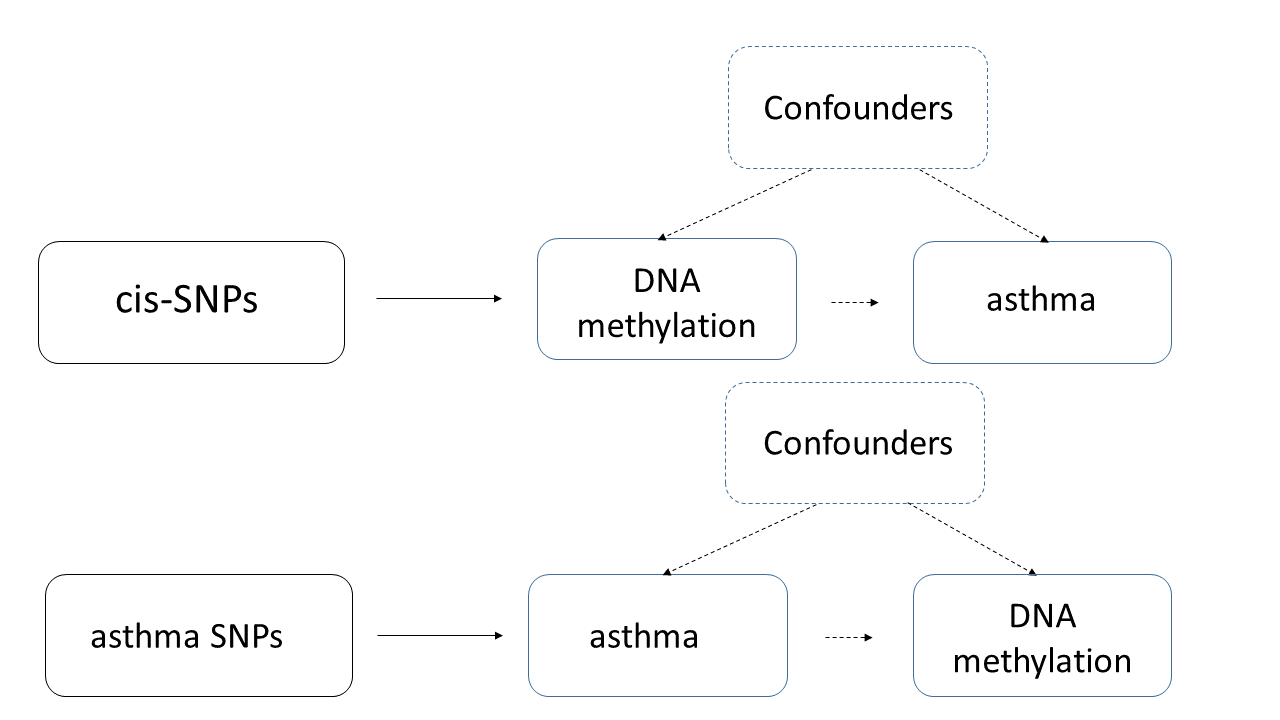


Figure S 2 Two-sample Mendelian Randomization in the asthma to DNA methylation direction.


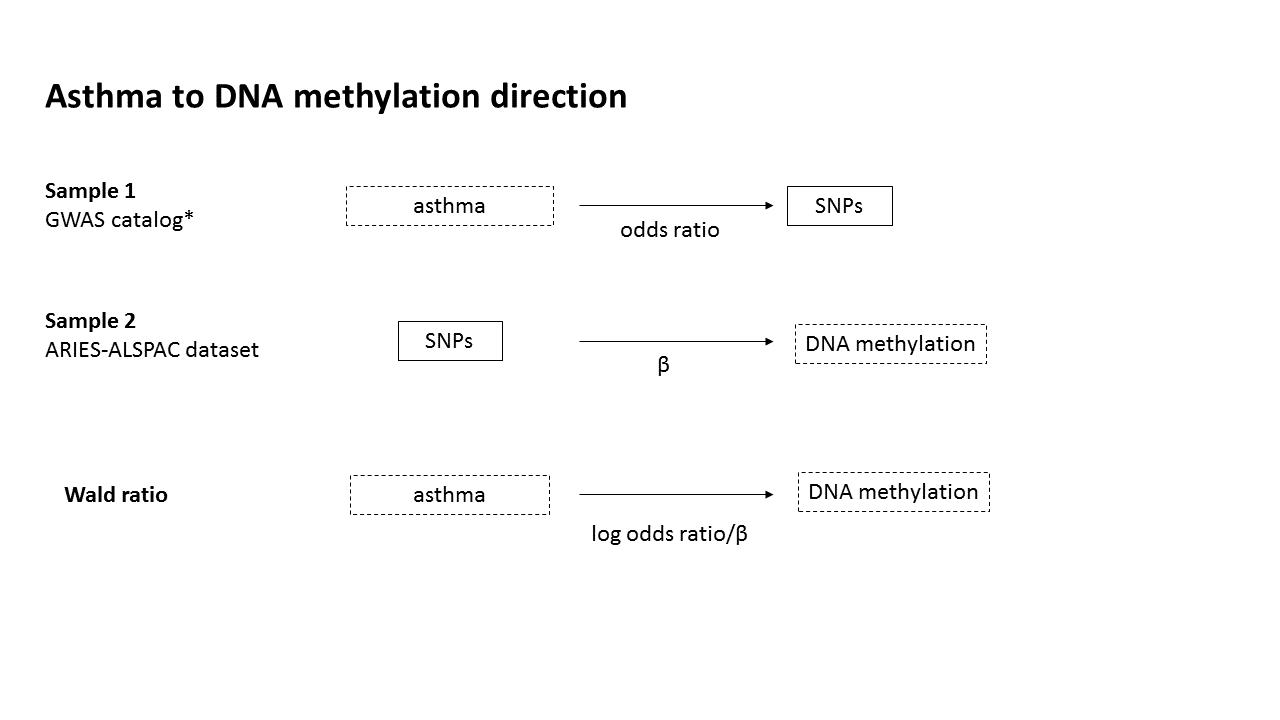


*GWAS catalog was used to source asthma SNPs instead of using the significant SNPs in the GABRIEL consortium, in order to allow more SNPs to be used as instruments and increase power.

Figure S 3 Two-sample Mendelian randomization in the DNA methylation to asthma direction.


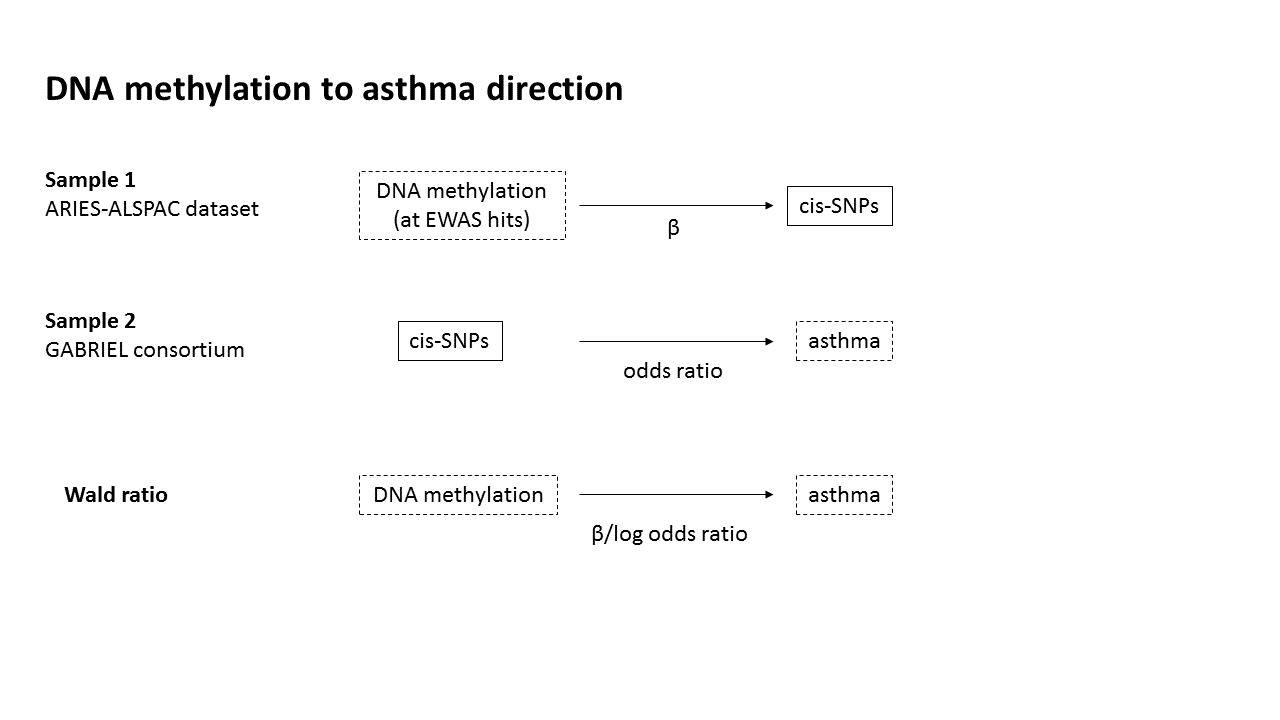


Figure S 4 Box-plot of the derived proportions of the different cell counts at 7.5 years in individuals with wheeze and individuals with no wheeze at 7.5 years.

**
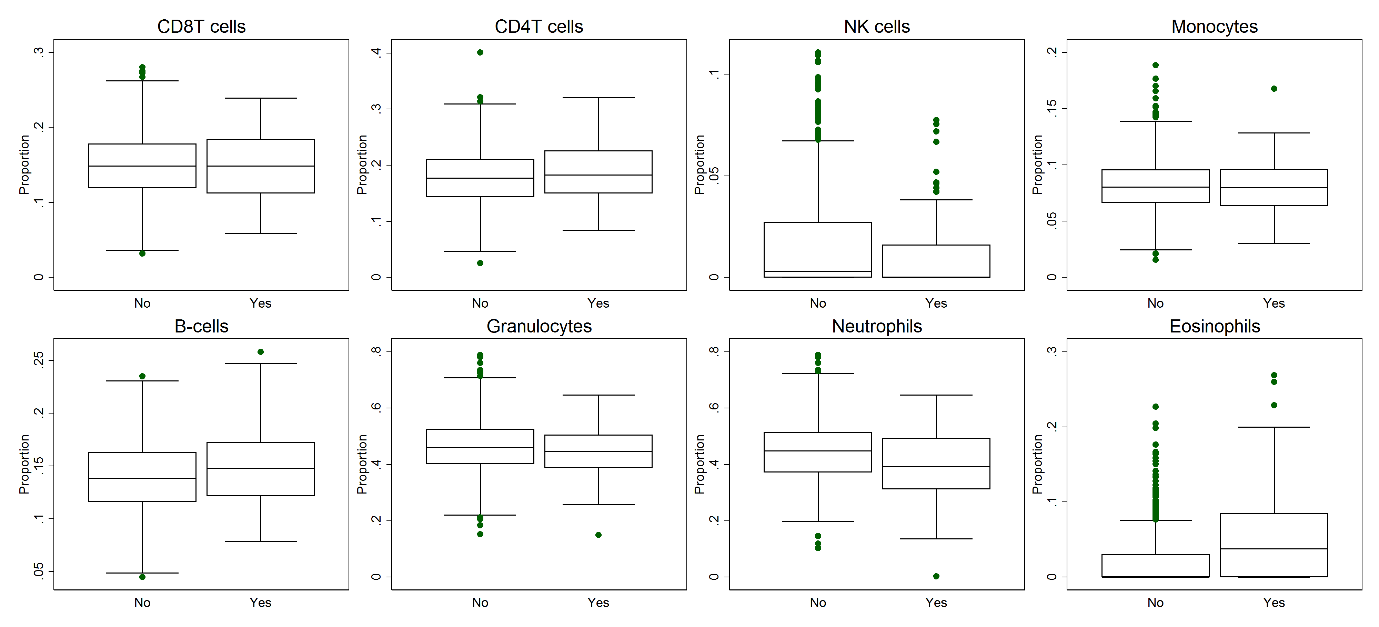
**

Table S 3 Numbers of atopic and non-atopic individuals by each asthma/wheeze exposure assessed in EWAS.

| **Methylation (outcome)** | **Asthma/ wheeze**  **(exposure)** | **Status** | **N in EWAS** | **N with skin prick test** | **Atopic N(%)*** |
| --- | --- | --- | --- | --- | --- |
| 7.5 years | Current asthma at 7.5 years | Cases | 149 | 134 | 60 (45%) |
|  |  | Controls | 632 | 562 | 94 (17%) |
|  | Current wheeze at 7.5 years | Cases | 111 | 100 | 53 (53%) |
|  |  | Controls | 742 | 657 | 117 (18%) |
| 16.5 years | Ever asthma at 16.5 years | Cases | 194 | 171 | 71 (41%) |
|  |  | Controls | 554 | 483 | 77 (16%) |
|  | Ever wheeze at 18.5 years | Cases | 204 | 176 | 71 (40%) |
|  |  | Controls | 554 | 487 | 78 (16%) |
|  | Current asthma at 16.5 years | Cases | 184 | 160 | 61 (38%) |
|  |  | Controls | 427 | 378 | 54 (14%) |

* Atopic status defined in a subset of individuals as a positive reaction to one or more of grass, pollen, house dust mite and cat at 7.5 years of age.

Table S 4 Associations of current asthma at 7.5 years with cell counts stratified by atopic status at 7.5 years. Atopic status is defined in a subset of individuals from the EWAS of current asthma as a positive reaction to one or more of grass, pollen, house dust mite and cat at 7.5 years of age.

|  | **Atopic (N=154)** | | **Non-atopic (N=542)** | |
| --- | --- | --- | --- | --- |
| **Cell type** | **Change in proportion* [95% CI]** | **P-value** | **Change in proportion* [95% CI]** | **P-value** |
| Monocytes | -0.005[-0.013,0.003] | 0.206 | 0.002[-0.004,0.008] | 0.510 |
| CD8T | -0.008[-0.022,0.005] | 0.236 | 0.007[-0.004,0.018] | 0.203 |
| CD4T | 0.011[-0.006,0.028] | 0.195 | 0.005[-0.007,0.017] | 0.432 |
| B-cells | 0.002[-0.009,0.013] | 0.760 | 0.015[0.007,0.023] | <0.001 |
| NK cells | 0.001[-0.007,0.008] | 0.928 | -0.004[-0.009,0.002] | 0.177 |
| Granulocytes | 0.001[-0.029,0.029] | 0.981 | -0.023[-0.046,-0.001] | 0.053 |
| Neutrophils | -0.032[-0.067,0.003] | 0.070 | -0.039[-0.065,-0.014] | 0.004 |
| Eosinophils | 0.032[0.016,0.047] | <0.001 | 0.016[0.007,0.025] | <0.001 |

* change in proportion for individuals with asthma compared to individuals without asthma.

Table S 5 Effect of different methods of eosinophil outlier removal on the association of eosinophil cell counts at 7.5 years with asthma status at 7.5 years of age.

| **Outlier Method** | **Difference in eosinophil proportion* [95% CI]** | **P-value** | **N removed (Female/ Male)** |
| --- | --- | --- | --- |
| No outlier removal | 0.026 [0.018,0.033] | <0.0001 | - |
| Tukey’s (stringent)† | 0.010 [0.005,0.015] | <0.0001 | 57 (18/39) |
| Tukey’s (relaxed) † | 0.018 [0.011,0.024] | <0.0001 | 14(4/10) |

* Difference in eosinophil proportion between individuals with asthma compared to individuals without asthma.

†Tukey’s (stringent) method removes eosinophil count outliers if value>1.5*IQR, Tukey’s (relaxed) method removes eosinophil count outliers if value >3*IQR.

Table S 6 Effect of eosinophil outlier removal on eosinophil count associations with asthma status at 7.5 years of age stratified by sex.

|  | **Male (N= 396)** | | | **Female (N=385)** | | |
| --- | --- | --- | --- | --- | --- | --- |
| **Outlier Method** | **Difference in eosinophil proportion* [95% CI]** | **P** | **N males removed** | **Difference in eosinophil proportion* [95% CI]** | **P** | **N females removed** |
| No outlier removal | 0.032 [0.022,0.041] | <0.001 | - | 0.015 [0.005,0.025] | 0.004 | - |
| Tukey’s (stringent)† | 0.028 [0.019,0.038] | <0.001 | 27 | 0.004 [-0.003,0.011] | 0.147 | 35 |
| Tukey’s (relaxed)† | 0.019 [0.011,0.026] | <0.001 | 3 | 0.004 [-0.001,0.009] | 0.281 | 15 |

* Difference in eosinophil proportion between individuals with asthma compared to individuals without asthma.

†Tukey’s (stringent) method removes eosinophil count outliers if value>1.5*IQR, Tukey’s (relaxed) method removes eosinophil count outliers if value >3*IQR.

Figure S 5 Manhattan plots of all EWAS models with FDR adjusted P-value <0.05 cut-off line in green and Bonferroni significance threshold<0.05 in red. Manhattan plots for EWAS are only shown if associations (FDR<0.05) existed.


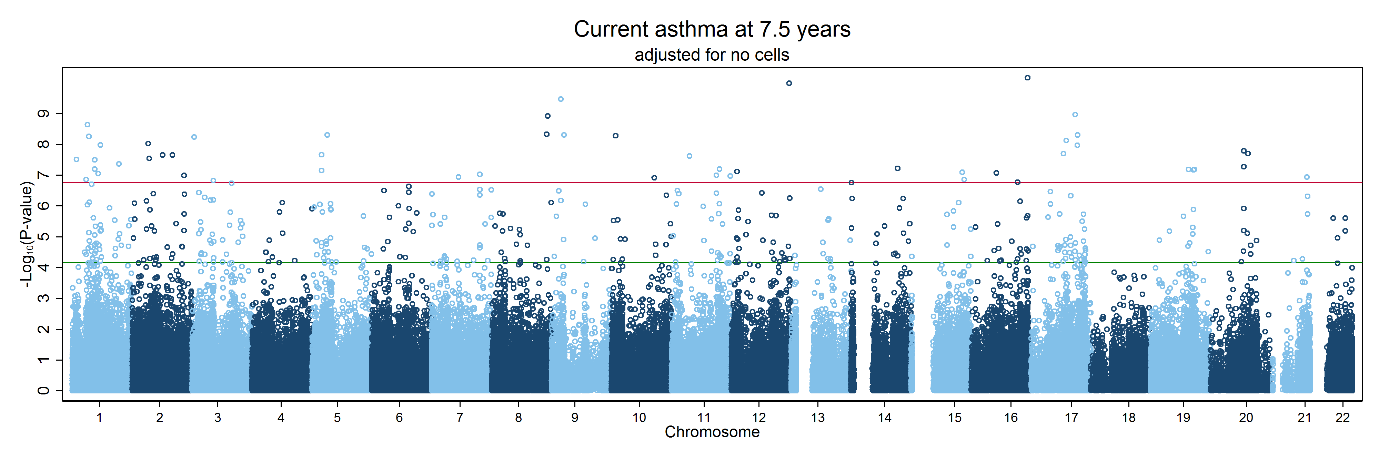

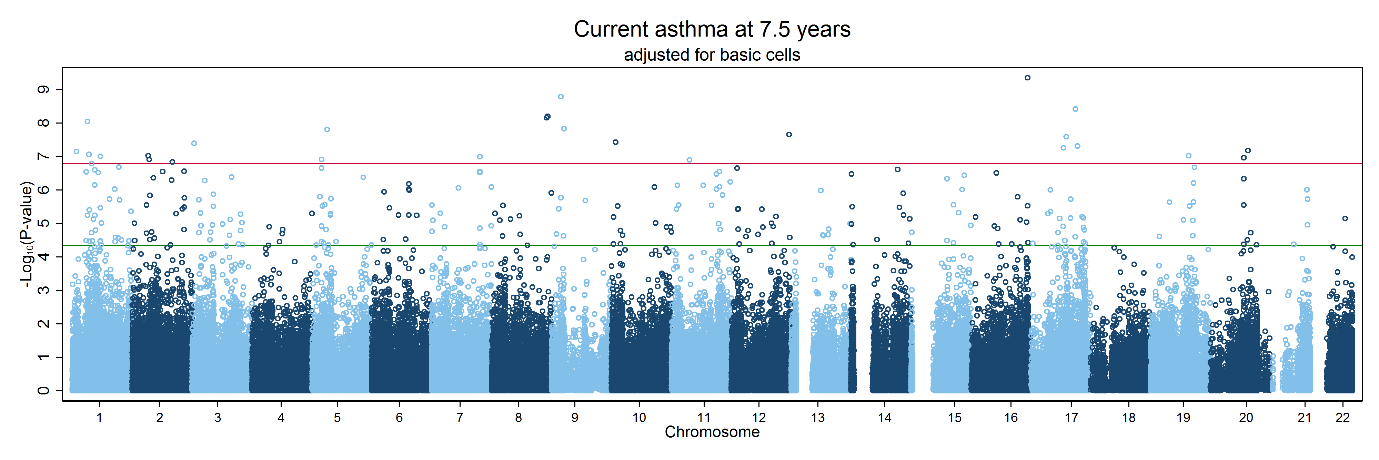


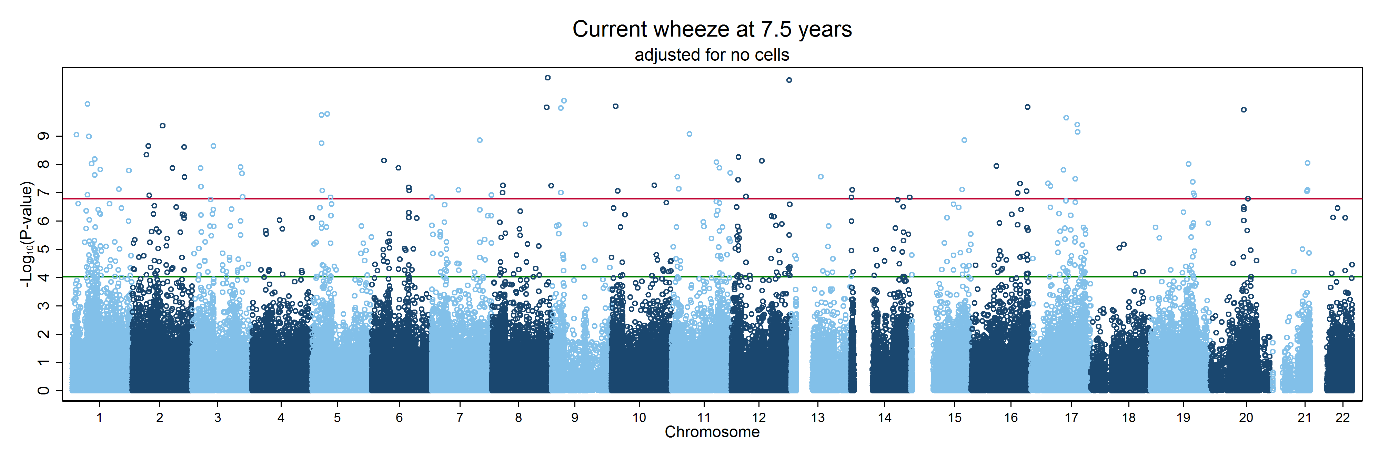

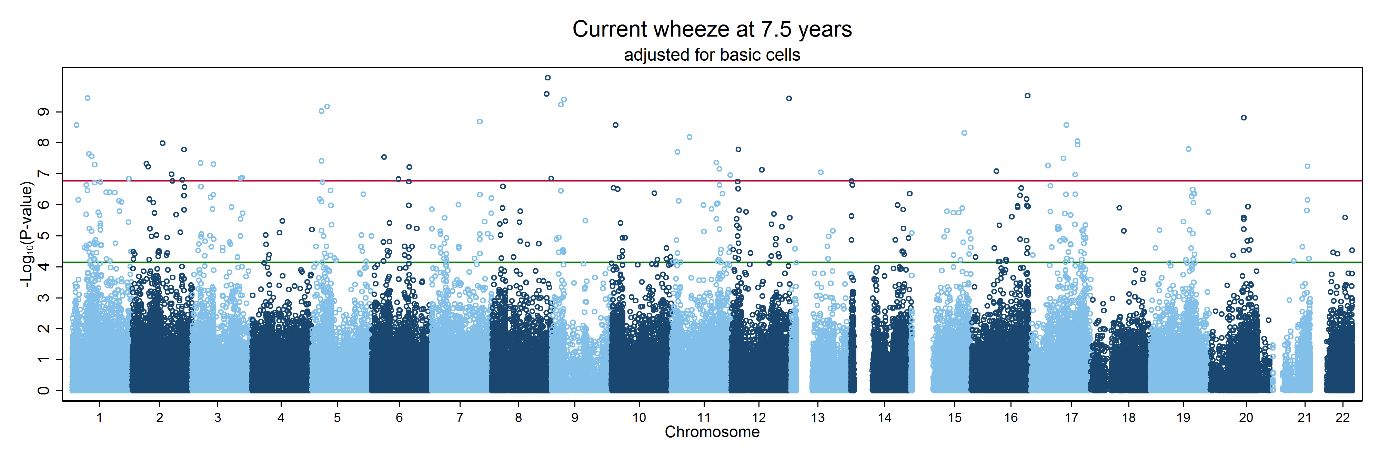


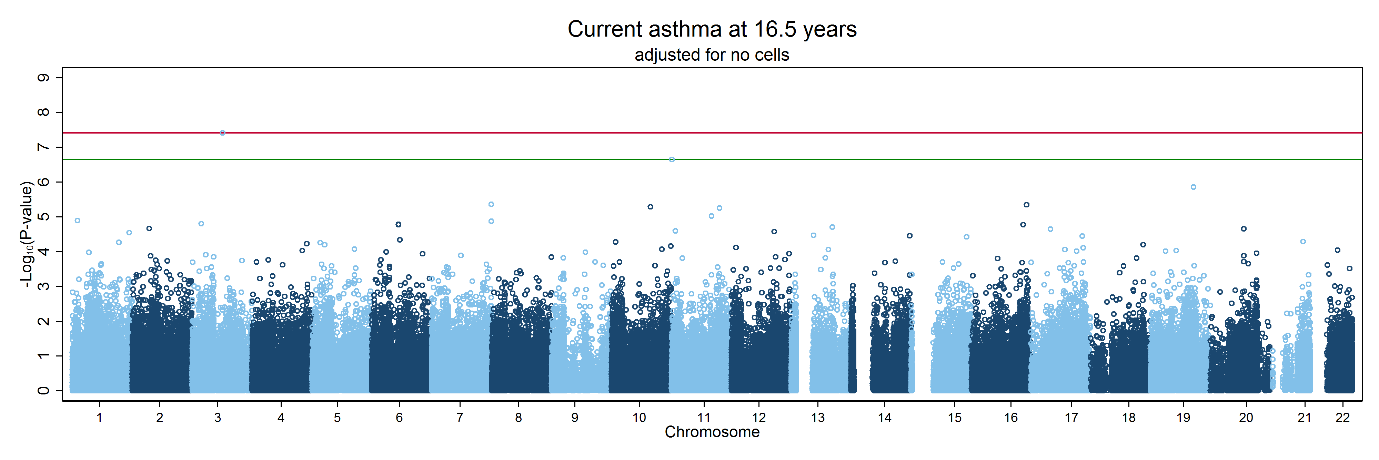

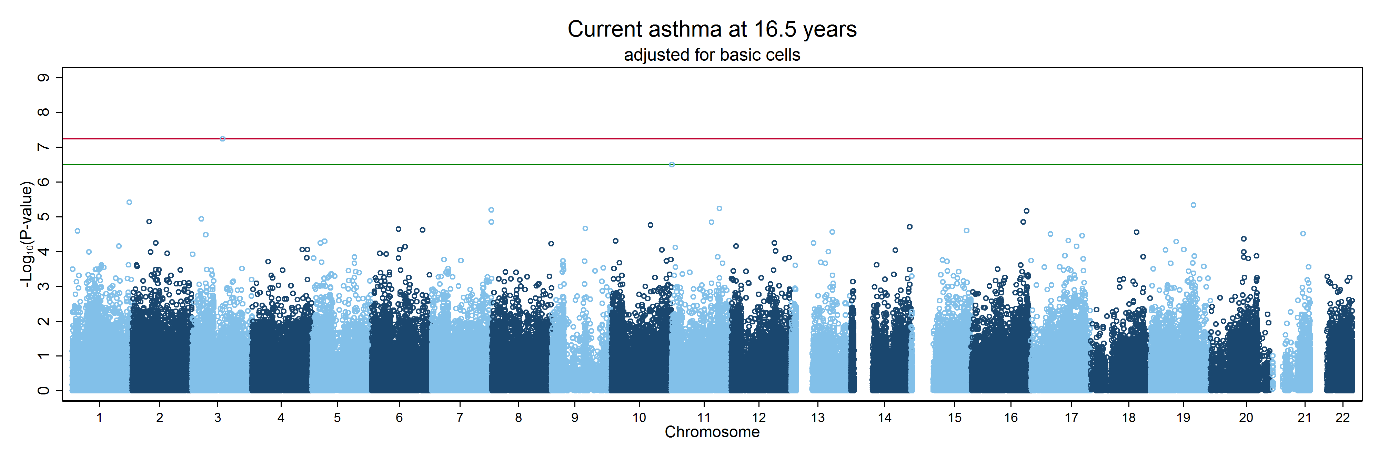


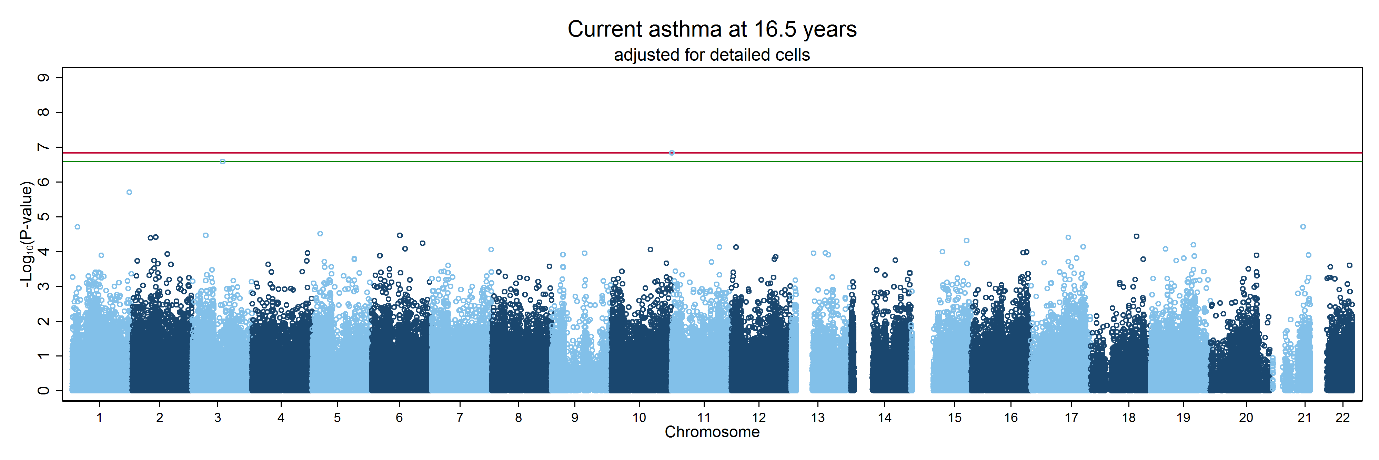


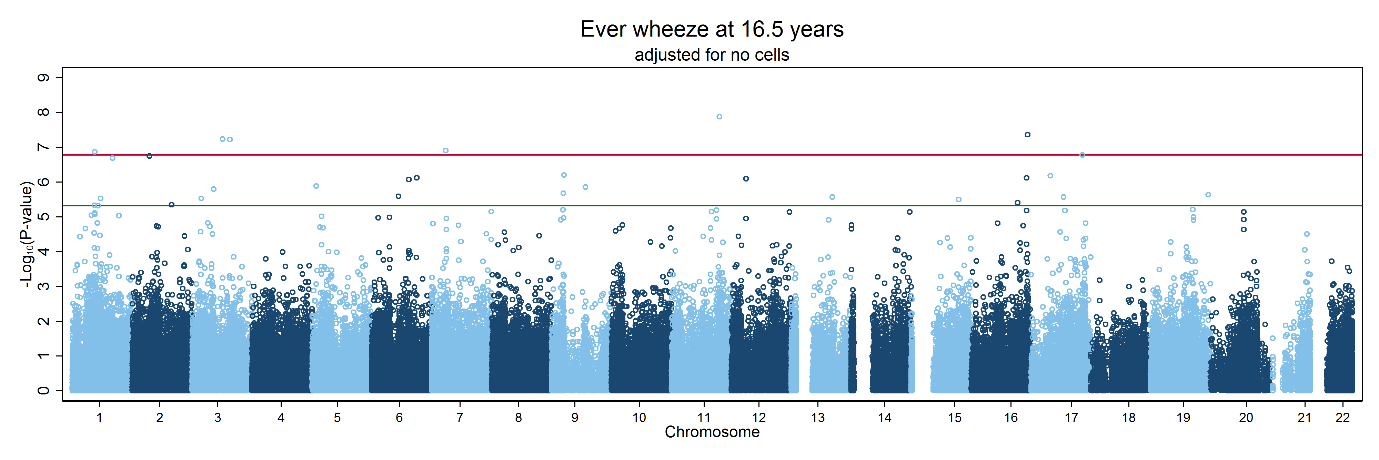


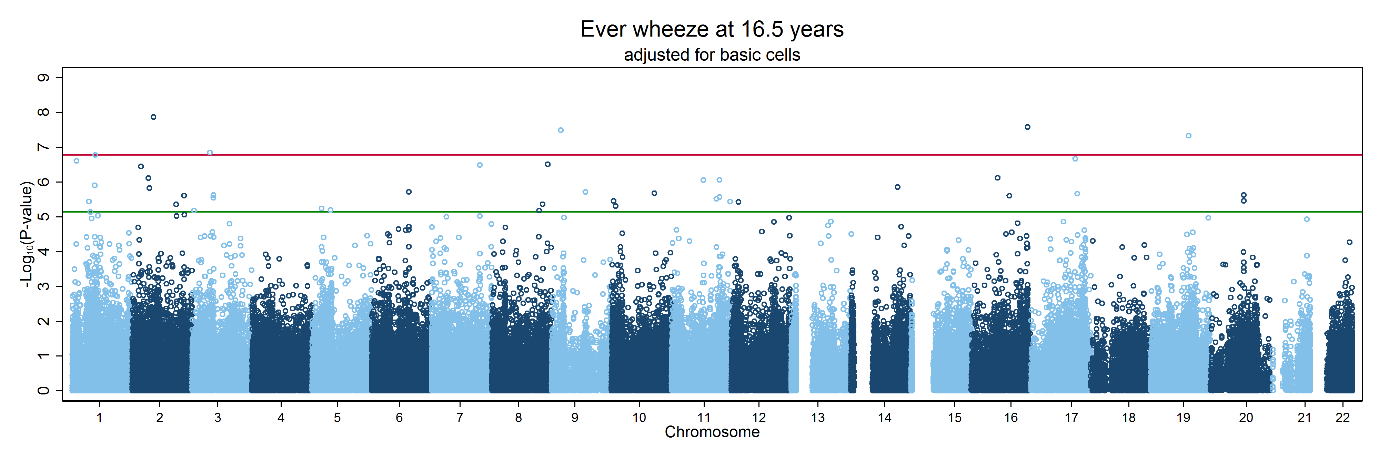


Figure S 6 Quantile-quantile (Q-Q) plots with genomic inflation factor (Lambda – λ) for each EWAS model.


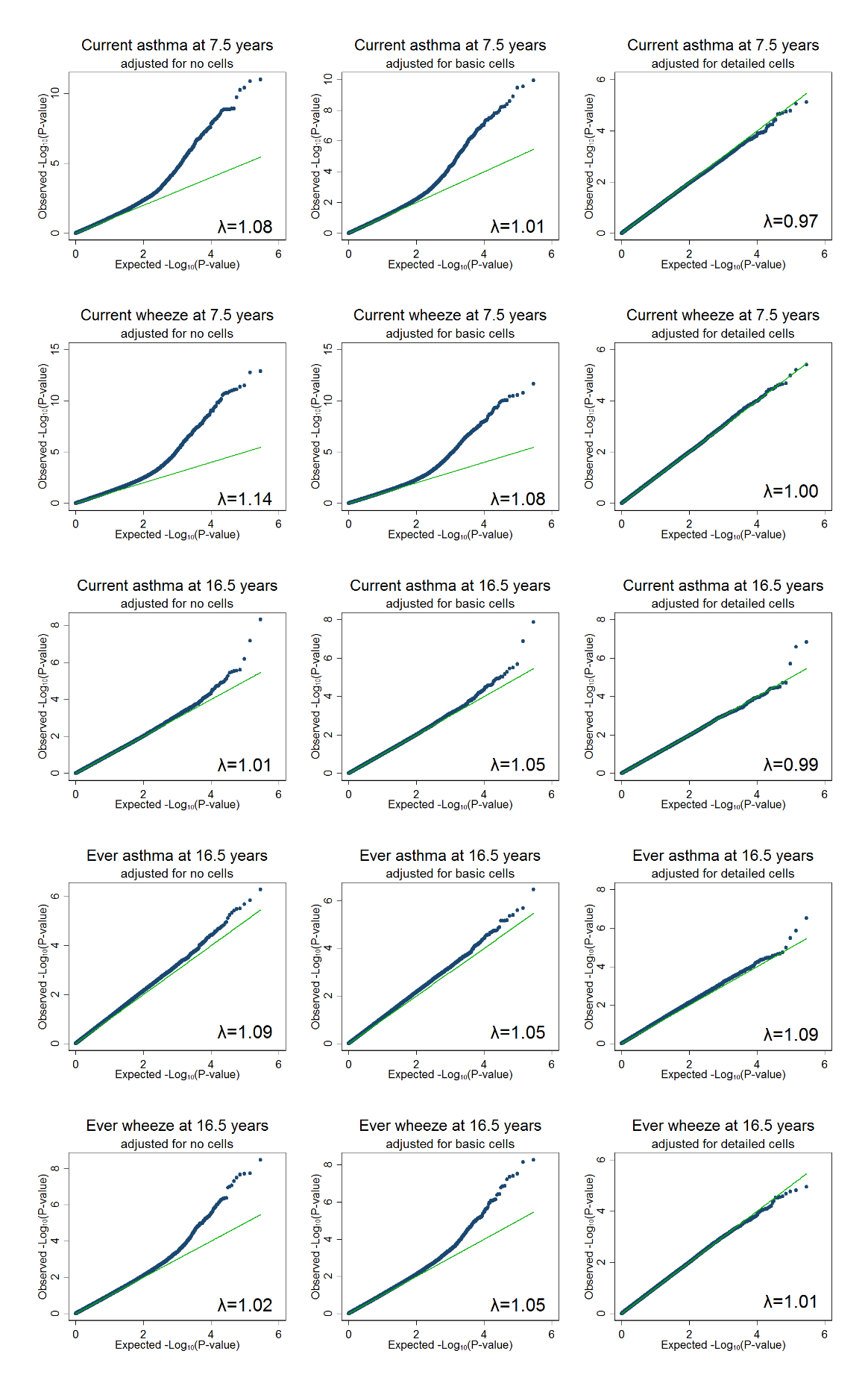


Figure S 7 Quantile-quantile (Q-Q) plots with genomic inflation factor (Lambda – λ) for each longitudinal EWAS model.

**
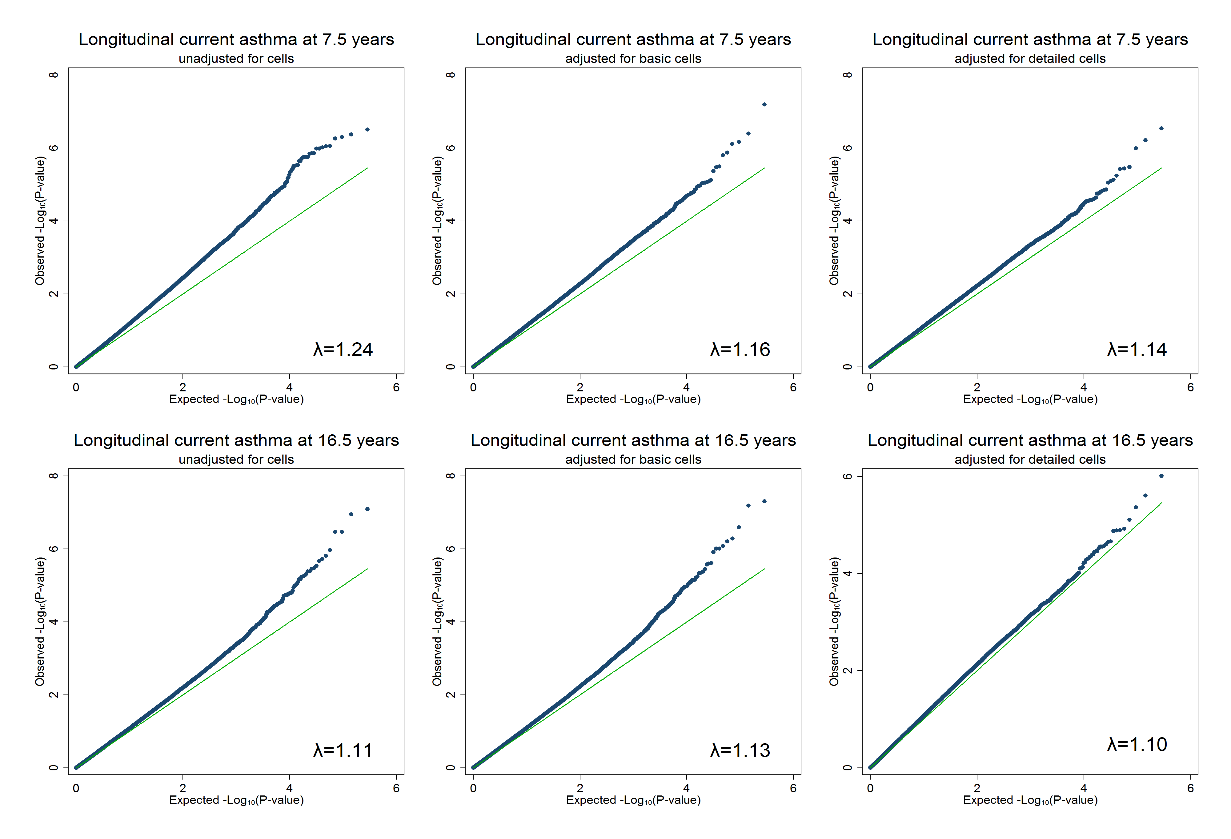
**

Table S 7 GO pathways that are functionally enriched (enrichment P-value<0.05) in the genes annotated to hit CpGs from the EWAS of current asthma at 7.5 years adjusted for basic cells.

| **Category** | **Term** | **Count** | **%** | **P-value** | **Fold enrichment** |
| --- | --- | --- | --- | --- | --- |
| GOTERM_BP_FAT | GO:0032673~regulation of interleukin-4 production | 5 | 2.6 | 2.23E-04 | 16.4 |
| GOTERM_BP_FAT | GO:0006928~movement of cell or subcellular component | 38 | 19.8 | 2.33E-04 | 1.8 |
| GOTERM_BP_FAT | GO:0040011~locomotion | 36 | 18.8 | 2.84E-04 | 1.9 |
| GOTERM_BP_FAT | GO:0032633~interleukin-4 production | 5 | 2.6 | 2.91E-04 | 15.3 |
| GOTERM_BP_FAT | GO:0072677~eosinophil migration | 4 | 2.1 | 4.66E-04 | 25.4 |
| GOTERM_BP_FAT | GO:0006022~aminoglycan metabolic process | 9 | 4.7 | 5.93E-04 | 4.8 |
| GOTERM_BP_FAT | GO:2000418~positive regulation of eosinophil migration | 3 | 1.6 | 6.43E-04 | 71.3 |
| GOTERM_BP_FAT | GO:0001819~positive regulation of cytokine production | 13 | 6.8 | 9.70E-04 | 3.1 |
| GOTERM_BP_FAT | GO:0051674~localization of cell | 27 | 14.1 | 1.06E-03 | 2.0 |
| GOTERM_BP_FAT | GO:0048870~cell motility | 27 | 14.1 | 1.06E-03 | 2.0 |
| GOTERM_BP_FAT | GO:0002274~myeloid leukocyte activation | 8 | 4.2 | 1.09E-03 | 5.0 |
| GOTERM_BP_FAT | GO:0032753~positive regulation of interleukin-4 production | 4 | 2.1 | 1.12E-03 | 19.0 |
| GOTERM_BP_FAT | GO:0032653~regulation of interleukin-10 production | 5 | 2.6 | 1.13E-03 | 10.8 |
| GOTERM_BP_FAT | GO:0042093~T-helper cell differentiation | 5 | 2.6 | 1.13E-03 | 10.8 |
| GOTERM_BP_FAT | GO:0002294~CD4-positive, alpha-beta T cell differentiation involved in immune response | 5 | 2.6 | 1.34E-03 | 10.3 |
| GOTERM_BP_FAT | GO:0032613~interleukin-10 production | 5 | 2.6 | 1.45E-03 | 10.1 |
| GOTERM_BP_FAT | GO:0002293~alpha-beta T cell differentiation involved in immune response | 5 | 2.6 | 1.45E-03 | 10.1 |
| GOTERM_BP_FAT | GO:0002287~alpha-beta T cell activation involved in immune response | 5 | 2.6 | 1.45E-03 | 10.1 |
| GOTERM_BP_FAT | GO:0051156~glucose 6-phosphate metabolic process | 4 | 2.1 | 1.49E-03 | 17.3 |
| GOTERM_BP_FAT | GO:0042116~macrophage activation | 5 | 2.6 | 0.001567 | 9.9 |
| GOTERM_BP_FAT | GO:2000416~regulation of eosinophil migration | 3 | 1.6 | 0.001585 | 47.6 |
| GOTERM_BP_FAT | GO:0030203~glycosaminoglycan metabolic process | 8 | 4.2 | 0.002082 | 4.5 |
| GOTERM_BP_FAT | GO:1903510~mucopolysaccharide metabolic process | 7 | 3.6 | 0.002266 | 5.2 |
| GOTERM_BP_FAT | GO:0002292~T cell differentiation involved in immune response | 5 | 2.6 | 0.002426 | 8.8 |
| GOTERM_BP_FAT | GO:0043367~CD4-positive, alpha-beta T cell differentiation | 5 | 2.6 | 0.002958 | 8.3 |
| GOTERM_BP_FAT | GO:0055082~cellular chemical homeostasis | 16 | 8.3 | 0.003006 | 2.4 |
| GOTERM_BP_FAT | GO:0001817~regulation of cytokine production | 15 | 7.8 | 0.003127 | 2.5 |
| GOTERM_BP_FAT | GO:0051270~regulation of cellular component movement | 18 | 9.4 | 0.003177 | 2.2 |
| GOTERM_BP_FAT | GO:0002573~myeloid leukocyte differentiation | 8 | 4.2 | 0.003858 | 4.0 |
| GOTERM_BP_FAT | GO:0030334~regulation of cell migration | 16 | 8.3 | 0.004113 | 2.3 |
| GOTERM_BP_FAT | GO:0042092~type 2 immune response | 4 | 2.1 | 0.004455 | 11.9 |
| GOTERM_BP_FAT | GO:0035710~CD4-positive, alpha-beta T cell activation | 5 | 2.6 | 0.004497 | 7.4 |
| GOTERM_BP_FAT | GO:0002676~regulation of chronic inflammatory response | 3 | 1.6 | 0.005616 | 25.9 |
| GOTERM_BP_FAT | GO:0002366~leukocyte activation involved in immune response | 8 | 4.2 | 0.00595 | 3.7 |
| GOTERM_BP_FAT | GO:0002263~cell activation involved in immune response | 8 | 4.2 | 0.006301 | 3.7 |
| GOTERM_BP_FAT | GO:0097285~cell-type specific apoptotic process | 12 | 6.3 | 0.006303 | 2.6 |
| GOTERM_BP_FAT | GO:2000145~regulation of cell motility | 16 | 8.3 | 0.006885 | 2.2 |
| GOTERM_BP_FAT | GO:0006024~glycosaminoglycan biosynthetic process | 6 | 3.1 | 0.006917 | 5.0 |
| GOTERM_BP_FAT | GO:0001816~cytokine production | 15 | 7.8 | 0.006969 | 2.2 |
| GOTERM_BP_FAT | GO:0006023~aminoglycan biosynthetic process | 6 | 3.1 | 0.007172 | 5.0 |
| GOTERM_BP_FAT | GO:0019725~cellular homeostasis | 17 | 8.9 | 0.007216 | 2.1 |
| GOTERM_BP_FAT | GO:0042592~homeostatic process | 28 | 14.6 | 0.007786 | 1.7 |
| GOTERM_BP_FAT | GO:0002830~positive regulation of type 2 immune response | 3 | 1.6 | 0.007856 | 21.9 |
| GOTERM_BP_FAT | GO:0030003~cellular cation homeostasis | 13 | 6.8 | 0.007973 | 2.4 |
| GOTERM_BP_FAT | GO:0048812~neuron projection morphogenesis | 18 | 9.4 | 0.009078 | 2.0 |
| GOTERM_BP_FAT | GO:0001774~microglial cell activation | 3 | 1.6 | 0.009102 | 20.4 |
| GOTERM_BP_FAT | GO:0055080~cation homeostasis | 14 | 7.3 | 0.009376 | 2.2 |
| GOTERM_BP_FAT | GO:0006873~cellular ion homeostasis | 13 | 6.8 | 0.009607 | 2.3 |
| GOTERM_BP_FAT | GO:0009605~response to external stimulus | 41 | 21.4 | 0.009624 | 1.5 |
| GOTERM_BP_FAT | GO:0016477~cell migration | 22 | 11.5 | 0.009642 | 1.8 |
| GOTERM_BP_FAT | GO:0030030~cell projection organization | 27 | 14.1 | 0.009677 | 1.7 |
| GOTERM_BP_FAT | GO:0006915~apoptotic process | 30 | 15.6 | 0.009763 | 1.6 |
| GOTERM_BP_FAT | GO:0045064~T-helper 2 cell differentiation | 3 | 1.6 | 0.010431 | 19.0 |
| GOTERM_BP_FAT | GO:0098771~inorganic ion homeostasis | 14 | 7.3 | 0.011103 | 2.2 |
| GOTERM_BP_FAT | GO:0046632~alpha-beta T cell differentiation | 5 | 2.6 | 0.011633 | 5.7 |
| GOTERM_BP_FAT | GO:0031175~neuron projection development | 21 | 10.9 | 0.011816 | 1.8 |
| GOTERM_BP_FAT | GO:0032693~negative regulation of interleukin-10 production | 3 | 1.6 | 0.01184 | 17.8 |
| GOTERM_BP_FAT | GO:0008643~carbohydrate transport | 7 | 3.6 | 0.012262 | 3.7 |
| GOTERM_BP_FAT | GO:0012501~programmed cell death | 31 | 16.1 | 0.01227 | 1.6 |
| GOTERM_BP_FAT | GO:1902105~regulation of leukocyte differentiation | 8 | 4.2 | 0.013205 | 3.2 |
| GOTERM_BP_FAT | GO:0002286~T cell activation involved in immune response | 5 | 2.6 | 0.013617 | 5.4 |
| GOTERM_BP_FAT | GO:0040012~regulation of locomotion | 16 | 8.3 | 0.013751 | 2.0 |
| GOTERM_BP_FAT | GO:0016265~death | 32 | 16.7 | 0.014138 | 1.5 |
| GOTERM_BP_FAT | GO:0008219~cell death | 32 | 16.7 | 0.014138 | 1.5 |
| GOTERM_BP_FAT | GO:0051047~positive regulation of secretion | 10 | 5.2 | 0.015426 | 2.6 |
| GOTERM_BP_FAT | GO:0015758~glucose transport | 6 | 3.1 | 0.016296 | 4.0 |
| GOTERM_BP_FAT | GO:0048878~chemical homeostasis | 19 | 9.9 | 0.016465 | 1.8 |
| GOTERM_BP_FAT | GO:0032674~regulation of interleukin-5 production | 3 | 1.6 | 0.01653 | 15.0 |
| GOTERM_BP_FAT | GO:0043066~negative regulation of apoptotic process | 17 | 8.9 | 0.016853 | 1.9 |
| GOTERM_BP_FAT | GO:0031016~pancreas development | 5 | 2.6 | 0.016976 | 5.1 |
| GOTERM_BP_FAT | GO:0008645~hexose transport | 6 | 3.1 | 0.017219 | 4.0 |
| GOTERM_BP_FAT | GO:0005975~carbohydrate metabolic process | 19 | 9.9 | 0.017394 | 1.8 |
| GOTERM_BP_FAT | GO:0015749~monosaccharide transport | 6 | 3.1 | 0.018174 | 3.9 |
| GOTERM_BP_FAT | GO:0016052~carbohydrate catabolic process | 6 | 3.1 | 0.018174 | 3.9 |
| GOTERM_BP_FAT | GO:0043069~negative regulation of programmed cell death | 17 | 8.9 | 0.018415 | 1.9 |
| GOTERM_BP_FAT | GO:0002285~lymphocyte activation involved in immune response | 6 | 3.1 | 0.018623 | 3.9 |
| GOTERM_BP_FAT | GO:0048666~neuron development | 22 | 11.5 | 0.019966 | 1.7 |
| GOTERM_BP_FAT | GO:0043011~myeloid dendritic cell differentiation | 3 | 1.6 | 0.020025 | 13.6 |
| GOTERM_BP_FAT | GO:0048858~cell projection morphogenesis | 20 | 10.4 | 0.020038 | 1.7 |
| GOTERM_BP_FAT | GO:0009117~nucleotide metabolic process | 14 | 7.3 | 0.020147 | 2.0 |
| GOTERM_BP_FAT | GO:0006796~phosphate-containing compound metabolic process | 44 | 22.9 | 0.020234 | 1.4 |
| GOTERM_BP_FAT | GO:0002682~regulation of immune system process | 26 | 13.5 | 0.020541 | 1.6 |
| GOTERM_BP_FAT | GO:1903706~regulation of hemopoiesis | 9 | 4.7 | 0.020756 | 2.6 |
| GOTERM_BP_FAT | GO:1903660~negative regulation of complement-dependent cytotoxicity | 2 | 1.0 | 0.020798 | 95.1 |
| GOTERM_BP_FAT | GO:2000422~regulation of eosinophil chemotaxis | 2 | 1.0 | 0.020798 | 95.1 |
| GOTERM_BP_FAT | GO:2000424~positive regulation of eosinophil chemotaxis | 2 | 1.0 | 0.020798 | 95.1 |
| GOTERM_BP_FAT | GO:0032601~connective tissue growth factor production | 2 | 1.0 | 0.020798 | 95.1 |
| GOTERM_BP_FAT | GO:1901657~glycosyl compound metabolic process | 10 | 5.2 | 0.021591 | 2.4 |
| GOTERM_BP_FAT | GO:0030217~T cell differentiation | 7 | 3.6 | 0.021732 | 3.2 |
| GOTERM_BP_FAT | GO:0007018~microtubule-based movement | 7 | 3.6 | 0.021732 | 3.2 |
| GOTERM_BP_FAT | GO:0030204~chondroitin sulfate metabolic process | 4 | 2.1 | 0.021746 | 6.7 |
| GOTERM_BP_FAT | GO:0006753~nucleoside phosphate metabolic process | 14 | 7.3 | 0.021761 | 2.0 |
| GOTERM_BP_FAT | GO:0071887~leukocyte apoptotic process | 5 | 2.6 | 0.022199 | 4.7 |
| GOTERM_BP_FAT | GO:0050728~negative regulation of inflammatory response | 5 | 2.6 | 0.022199 | 4.7 |
| GOTERM_BP_FAT | GO:0050714~positive regulation of protein secretion | 7 | 3.6 | 0.022656 | 3.2 |
| GOTERM_BP_FAT | GO:0050801~ion homeostasis | 14 | 7.3 | 0.022972 | 2.0 |
| GOTERM_BP_FAT | GO:0097529~myeloid leukocyte migration | 6 | 3.1 | 0.023517 | 3.7 |
| GOTERM_BP_FAT | GO:0032990~cell part morphogenesis | 20 | 10.4 | 0.023672 | 1.7 |
| GOTERM_BP_FAT | GO:0002521~leukocyte differentiation | 11 | 5.7 | 0.024148 | 2.3 |
| GOTERM_BP_FAT | GO:0045321~leukocyte activation | 15 | 7.8 | 0.024799 | 1.9 |
| GOTERM_BP_FAT | GO:0050654~chondroitin sulfate proteoglycan metabolic process | 4 | 2.1 | 0.024865 | 6.3 |
| GOTERM_BP_FAT | GO:1903532~positive regulation of secretion by cell | 9 | 4.7 | 0.025112 | 2.5 |
| GOTERM_BP_FAT | GO:0002544~chronic inflammatory response | 3 | 1.6 | 0.025788 | 11.9 |
| GOTERM_BP_FAT | GO:0002520~immune system development | 16 | 8.3 | 0.025796 | 1.8 |
| GOTERM_BP_FAT | GO:0030099~myeloid cell differentiation | 9 | 4.7 | 0.025896 | 2.5 |
| GOTERM_BP_FAT | GO:0055065~metal ion homeostasis | 12 | 6.3 | 0.027557 | 2.1 |
| GOTERM_BP_FAT | GO:0030206~chondroitin sulfate biosynthetic process | 3 | 1.6 | 0.027841 | 11.4 |
| GOTERM_BP_FAT | GO:0002828~regulation of type 2 immune response | 3 | 1.6 | 0.027841 | 11.4 |
| GOTERM_BP_FAT | GO:0006875~cellular metal ion homeostasis | 11 | 5.7 | 0.028129 | 2.2 |
| GOTERM_BP_FAT | GO:0002761~regulation of myeloid leukocyte differentiation | 5 | 2.6 | 0.028305 | 4.3 |
| GOTERM_BP_FAT | GO:0042981~regulation of apoptotic process | 24 | 12.5 | 0.029649 | 1.6 |
| GOTERM_BP_FAT | GO:0045622~regulation of T-helper cell differentiation | 3 | 1.6 | 0.029957 | 11.0 |
| GOTERM_BP_FAT | GO:0009611~response to wounding | 19 | 9.9 | 0.030284 | 1.7 |
| GOTERM_BP_FAT | GO:0046631~alpha-beta T cell activation | 5 | 2.6 | 0.030828 | 4.2 |
| GOTERM_BP_FAT | GO:0072521~purine-containing compound metabolic process | 12 | 6.3 | 0.030898 | 2.1 |
| GOTERM_BP_FAT | GO:0042097~interleukin-4 biosynthetic process | 2 | 1.0 | 0.031035 | 63.4 |
| GOTERM_BP_FAT | GO:0045402~regulation of interleukin-4 biosynthetic process | 2 | 1.0 | 0.031035 | 63.4 |
| GOTERM_BP_FAT | GO:0002215~defense response to nematode | 2 | 1.0 | 0.031035 | 63.4 |
| GOTERM_BP_FAT | GO:0097278~complement-dependent cytotoxicity | 2 | 1.0 | 0.031035 | 63.4 |
| GOTERM_BP_FAT | GO:0051091~positive regulation of sequence-specific DNA binding transcription factor activity | 7 | 3.6 | 0.031057 | 3.0 |
| GOTERM_BP_FAT | GO:0043067~regulation of programmed cell death | 24 | 12.5 | 0.031954 | 1.6 |
| GOTERM_BP_FAT | GO:0061621~canonical glycolysis | 3 | 1.6 | 0.032136 | 10.6 |
| GOTERM_BP_FAT | GO:0006735~NADH regeneration | 3 | 1.6 | 0.032136 | 10.6 |
| GOTERM_BP_FAT | GO:0061620~glycolytic process through glucose-6-phosphate | 3 | 1.6 | 0.032136 | 10.6 |
| GOTERM_BP_FAT | GO:0061615~glycolytic process through fructose-6-phosphate | 3 | 1.6 | 0.032136 | 10.6 |
| GOTERM_BP_FAT | GO:0061718~glucose catabolic process to pyruvate | 3 | 1.6 | 0.032136 | 10.6 |
| GOTERM_BP_FAT | GO:0022008~neurogenesis | 27 | 14.1 | 0.032437 | 1.5 |
| GOTERM_BP_FAT | GO:0051050~positive regulation of transport | 17 | 8.9 | 0.032676 | 1.7 |
| GOTERM_BP_FAT | GO:0050727~regulation of inflammatory response | 8 | 4.2 | 0.03349 | 2.6 |
| GOTERM_BP_FAT | GO:0060548~negative regulation of cell death | 17 | 8.9 | 0.033516 | 1.7 |
| GOTERM_BP_FAT | GO:0048534~hematopoietic or lymphoid organ development | 15 | 7.8 | 0.033721 | 1.8 |
| GOTERM_BP_FAT | GO:0072593~reactive oxygen species metabolic process | 7 | 3.6 | 0.033969 | 2.9 |
| GOTERM_BP_FAT | GO:0006026~aminoglycan catabolic process | 4 | 2.1 | 0.034296 | 5.6 |
| GOTERM_BP_FAT | GO:0002702~positive regulation of production of molecular mediator of immune response | 4 | 2.1 | 0.034296 | 5.6 |
| GOTERM_BP_FAT | GO:0043030~regulation of macrophage activation | 3 | 1.6 | 0.034374 | 10.2 |
| GOTERM_BP_FAT | GO:1903426~regulation of reactive oxygen species biosynthetic process | 4 | 2.1 | 0.035588 | 5.5 |
| GOTERM_BP_FAT | GO:0014009~glial cell proliferation | 3 | 1.6 | 0.036672 | 9.8 |
| GOTERM_BP_FAT | GO:0070227~lymphocyte apoptotic process | 4 | 2.1 | 0.036904 | 5.4 |
| GOTERM_BP_FAT | GO:0007166~cell surface receptor signaling pathway | 39 | 20.3 | 0.038355 | 1.4 |
| GOTERM_BP_FAT | GO:0000902~cell morphogenesis | 24 | 12.5 | 0.038932 | 1.5 |
| GOTERM_BP_FAT | GO:0051452~intracellular pH reduction | 3 | 1.6 | 0.039027 | 9.5 |
| GOTERM_BP_FAT | GO:0008045~motor neuron axon guidance | 3 | 1.6 | 0.039027 | 9.5 |
| GOTERM_BP_FAT | GO:0019693~ribose phosphate metabolic process | 11 | 5.7 | 0.039201 | 2.1 |
| GOTERM_BP_FAT | GO:0045582~positive regulation of T cell differentiation | 4 | 2.1 | 0.039611 | 5.3 |
| GOTERM_BP_FAT | GO:0006163~purine nucleotide metabolic process | 11 | 5.7 | 0.039645 | 2.1 |
| GOTERM_BP_FAT | GO:0051090~regulation of sequence-specific DNA binding transcription factor activity | 9 | 4.7 | 0.039774 | 2.3 |
| GOTERM_BP_FAT | GO:0055086~nucleobase-containing small molecule metabolic process | 14 | 7.3 | 0.03981 | 1.8 |
| GOTERM_BP_FAT | GO:1901699~cellular response to nitrogen compound | 14 | 7.3 | 0.03981 | 1.8 |
| GOTERM_BP_FAT | GO:0050650~chondroitin sulfate proteoglycan biosynthetic process | 3 | 1.6 | 0.041437 | 9.2 |
| GOTERM_BP_FAT | GO:0030851~granulocyte differentiation | 3 | 1.6 | 0.041437 | 9.2 |
| GOTERM_BP_FAT | GO:0009116~nucleoside metabolic process | 9 | 4.7 | 0.041938 | 2.3 |
| GOTERM_BP_FAT | GO:0009167~purine ribonucleoside monophosphate metabolic process | 7 | 3.6 | 0.042412 | 2.7 |
| GOTERM_BP_FAT | GO:1901135~carbohydrate derivative metabolic process | 22 | 11.5 | 0.042843 | 1.5 |
| GOTERM_BP_FAT | GO:0030097~hemopoiesis | 14 | 7.3 | 0.043234 | 1.8 |
| GOTERM_BP_FAT | GO:0043010~camera-type eye development | 8 | 4.2 | 0.043288 | 2.5 |
| GOTERM_BP_FAT | GO:0030098~lymphocyte differentiation | 8 | 4.2 | 0.043288 | 2.5 |
| GOTERM_BP_FAT | GO:0009126~purine nucleoside monophosphate metabolic process | 7 | 3.6 | 0.043382 | 2.7 |
| GOTERM_BP_FAT | GO:0030316~osteoclast differentiation | 4 | 2.1 | 0.043856 | 5.1 |
| GOTERM_BP_FAT | GO:0001773~myeloid dendritic cell activation | 3 | 1.6 | 0.043903 | 8.9 |
| GOTERM_BP_FAT | GO:1901653~cellular response to peptide | 11 | 5.7 | 0.044761 | 2.0 |
| GOTERM_BP_FAT | GO:0045637~regulation of myeloid cell differentiation | 6 | 3.1 | 0.044979 | 3.1 |
| GOTERM_BP_FAT | GO:0072524~pyridine-containing compound metabolic process | 5 | 2.6 | 0.045382 | 3.7 |
| GOTERM_BP_FAT | GO:0045851~pH reduction | 3 | 1.6 | 0.046422 | 8.6 |
| GOTERM_BP_FAT | GO:0042339~keratan sulfate metabolic process | 3 | 1.6 | 0.046422 | 8.6 |
| GOTERM_BP_FAT | GO:1901700~response to oxygen-containing compound | 25 | 13.0 | 0.04738 | 1.5 |
| GOTERM_BP_FAT | GO:0071417~cellular response to organonitrogen compound | 13 | 6.8 | 0.047468 | 1.9 |
| GOTERM_BP_FAT | GO:0051092~positive regulation of NF-kappaB transcription factor activity | 5 | 2.6 | 0.047568 | 3.7 |
| GOTERM_BP_FAT | GO:0032868~response to insulin | 10 | 5.2 | 0.048716 | 2.1 |
| GOTERM_BP_FAT | GO:0043370~regulation of CD4-positive, alpha-beta T cell differentiation | 3 | 1.6 | 0.048992 | 8.4 |
| GOTERM_BP_FAT | GO:0006007~glucose catabolic process | 3 | 1.6 | 0.048992 | 8.4 |
| GOTERM_BP_FAT | GO:0070887~cellular response to chemical stimulus | 39 | 20.3 | 0.049265 | 1.3 |
| GOTERM_BP_FAT | GO:0046128~purine ribonucleoside metabolic process | 8 | 4.2 | 0.049857 | 2.4 |

Table S 8 KEGG pathways that are functionally enriched in genes annotated to the hit CpGS from the EWAS of current asthma at 7.5 years adjusted for basic cells.

| **Category** | **Term** | **Count** | **%** | **P-value** | **Fold enrichment** |
| --- | --- | --- | --- | --- | --- |
| KEGG_PATHWAY | hsa00520:Amino sugar and nucleotide sugar metabolism | 5 | 2.6 | 0.002079 | 9.0 |
| KEGG_PATHWAY | hsa05310:Asthma | 4 | 2.1 | 0.004748 | 11.5 |
| KEGG_PATHWAY | hsa00603:Glycosphingolipid biosynthesis - globo series | 3 | 1.6 | 0.011602 | 17.8 |
| KEGG_PATHWAY | hsa00604:Glycosphingolipid biosynthesis - ganglio series | 3 | 1.6 | 0.013284 | 16.6 |
| KEGG_PATHWAY | hsa01200:Carbon metabolism | 5 | 2.6 | 0.043983 | 3.7 |
| KEGG_PATHWAY | hsa00524:Butirosin and neomycin biosynthesis | 2 | 1.0 | 0.058115 | 33.2 |
| KEGG_PATHWAY | hsa05222:Small cell lung cancer | 4 | 2.1 | 0.080162 | 3.9 |
| KEGG_PATHWAY | hsa04973:Carbohydrate digestion and absorption | 3 | 1.6 | 0.088745 | 5.9 |

**Mendelian randomization**

**Power calculations**

For the power calculation in the asthma to DNA methylation direction of the MR, proportion of variance explained (PVE) for each asthma SNP used as an IV was calculated from summary statistics (Table S 8) and based on the method reported previously [6, 7]. Briefly, PVE was calculated using the following formula:


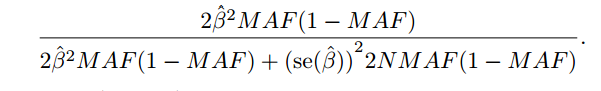


Where MAF is the minor/effect allele frequency of each SNP, N is the sample size of the GABRIEL GWAS of asthma (26475), β is the beta reported for the asthma-SNP effect and se is the standard error of the association. For the power calculation, the sum of the PVEs for each of the IVs was used.

For the power calculation, β_OLS_ was calculated as average absolute beta of the association of the 302 CpG sites tested. In the DNA methylation to asthma direction of the MR, the average of the absolute R-squared values for the cis-SNPs was used (Table S 9).

In the asthma to DNA methylation direction we had a maximum 6% power to detect a 3% change in methylation, using a sample size of 1000 (ARIES-ALSPAC size), α=0.05 (Type-I error rate, not taking in to account multiple testing), β_yx_=0.03 (estimated causal effect of asthma on methylation), β_OLS_=0.03 (average observational association of asthma on methylation), R^2^_xz_=0.01 (sum of proportion variance explained by all asthma SNPs used as IVs), σ^2^_(x)_=0.09 (assuming 10% of sample are asthma cases) and σ^2^_(y)_=0.01 (based on average 0.1 standard deviation for methylation at each CpG).

In the DNA methylation to asthma direction we had a maximum 73% power to detect an effect using a same sample size of 26475 (GABRIEL GWAS size) and α=0.05 (Type-I error rate), K=0.1 (estimated proportion of asthma cases) and assuming an average OR=1.1 (effect on asthma per standard deviation of DNA methylation), R^2^_xz_=0.28 (average absolute R^2^ value observed between cis-SNPs and DNA methylation).

The power calculator used[8] assumes a single sample and does not take in to account the two different sample sizes arising from a two-sample MR approach. We have used the outcome sample size in each direction, where the SNP-outcome effect was estimated. Power may hence be overestimated in the DNA methylation to asthma direction and underestimated power in the asthma to DNA methylation direction.

**Asthma IVs**

We performed two-sample MR in the asthma to DNA methylation direction using the TwoSampleMR R package available as part of the MRBase platform. To define Instrumental Variables (IVs) for asthma we performed a search on the GWAS catalog [9] for ‘asthma’ and “asthma(childhood onset)”. We filtered by a p-value threshold of <5e-08. Entries where the risk allele, beta or standard error was missing were removed, as well as entries that did not have additional required information for the TwoSampleMR package. Any entries that contained SNPs that were duplicated in the list were removed. We performed LD clumping to remove SNPs that were in strong LD with each other. SNPs in LD are far more likely to proxy the same genetic effect and violate the assumption of independent IVs in MR. We used an R^2^ LD cut-off of 0.1. A total of 6 SNPs remained after our exclusions that we used as asthma IVs (Table S 9).

Table S 9 List of SNPs used as IVs of asthma in two-sample Mendelian randomization.

| **SNP** | **Effect allele** | **Other allele** | **Beta** | **EAF*** | **P-value** | **SE** | **PVE†** | **Gene** | **Study** |
| --- | --- | --- | --- | --- | --- | --- | --- | --- | --- |
| rs13408661 | A | G | -0.207 | 0.16 | 1.00E-09 | 0.033 | 0.00146 | IL1RL1/  IL18R1 | Ramasamy et al[10] |
| rs1701704 | G | T | 0.174 | 0.18 | 2.00E-13 | 0.024 | 0.00207 | IKZF4 | Hirota et al[11] |
| rs4129267 | T | C | 0.086 | 0.37 | 2.00E-08 | 0.014 | 0.00142 | IL6R | Ferreira et al[12] |
| rs6967330 | A | G | 0.231 | 0.19 | 3.00E-14 | 0.031 | 0.00216 | CDHR3 | Bonnelykke et al[13] |
| rs7130588 | G | A | 0.086 | 0.34 | 2.00E-08 | 0.016 | 0.00105 | LRRC32 | Ferreira et al[12] |
| rs928413 | G | A | 0.215 | 0.28 | 9.00E-13 | 0.031 | 0.00184 | IL33 | Bonnelykke et al[13] |

* Effect allele frequency

†Proportion of variance explained, calculated from the summary statistics of the asthma-SNP associations

**DNA methylation IVs**

To define IVs that proxy methylation at the target CpGs, we performed a look-up of SNP-CpG associations using the ARIES-ALSPAC dataset. We searched for mQTLs (SNPs that are associated with methylation at specific CpG ) using the process outlined in mQTLdb (mqtldb.org)[14], a database that contains associations between SNPs and CpGs that draws on the data available in the ARIES project. We set a mQTL distance of 1Mb flanking and restricted the search to the childhood time-point at approximately 7.5 years of age for mQTLs of the 302 CpGs from the EWAS of asthma at 7.5 years and restricted to the adolescent time-point for the mQTL search of the 2 CpGs from the EWAS of asthma at 16.5 years. We removed SNPs that were due to insertions or deletions (indels) and SNPs that were in trans. A large proportion of the cis-SNPs were not found in the GABRIEL consortium summary dataset as there was no SNP imputation in the original GWAS study. As a result, 33 CpGs were found to have cis-SNPs available in the GABRIEL GWAS dataset. Two of the cis-SNPs had a beta and standard error of zero in the GABRIEL dataset and were excluded. We selected 1 cis-SNP per CpG as many of the cis-SNPs in each CpG were in strong LD with each other. Including cis-SNPs that are in strong LD with each other violates the independent IVs assumption of MR. Since only one SNP IV was used per CpG we calculated Wald ratios to determine the effect.

Table S 10 List of cis-SNPs used as IVs of methylation in two-sample Mendelian Randomization with the SNP-methylation effects.

| **CpG** | **SNP** | **A1** | **A2** | **Beta** | **SE** | **P-value** | **Frequency** | **r^2^** | **Gene** |
| --- | --- | --- | --- | --- | --- | --- | --- | --- | --- |
| cg01766943 | rs7608414 | A | G | 0.057 | 0.003 | 8.30E-56 | 0.230 | 0.506 | SPTBN1 |
| cg02246992 | rs2433 | C | T | -0.074 | 0.008 | 5.72E-18 | 0.099 | -0.292 | ATAD5 |
| cg02741985 | rs6502057 | A | G | 0.025 | 0.004 | 2.51E-10 | 0.390 | 0.216 | CCDC57 |
| cg03329755 | rs6913892 | A | G | 0.037 | 0.003 | 4.55E-27 | 0.160 | 0.360 | RPS6KA2 |
| cg03759239 | rs11576197 | G | A | -0.018 | 0.002 | 1.05E-14 | 0.495 | -0.262 | RGS13 |
| cg03935956 | rs9927961 | G | A | 0.037 | 0.004 | 2.95E-23 | 0.190 | 0.333 | GGA2 |
| cg04111761 | rs1491961 | T | C | 0.019 | 0.003 | 2.61E-08 | 0.272 | 0.190 | CCR3 |
| cg06216065 | rs17162330 | C | T | -0.034 | 0.003 | 7.04E-23 | 0.165 | -0.330 | ZDHHC18 |
| cg07307484 | rs8072531 | G | A | 0.010 | 0.002 | 2.15E-09 | 0.422 | 0.205 | MINK1 |
| cg07625783 | rs12046006 | C | T | -0.031 | 0.005 | 5.57E-09 | 0.099 | -0.199 | SLAMF8 |
| cg07955754 | rs2190245 | T | G | 0.010 | 0.001 | 2.52E-13 | 0.490 | 0.249 | TAS2R4 |
| cg10097651 | rs10147645 | G | A | -0.007 | 0.001 | 1.80E-10 | 0.074 | -0.218 |  |
| cg10498052 | rs4792750 | T | C | -0.012 | 0.002 | 2.94E-14 | 0.339 | -0.258 | NCRNA00188;C17orf76 |
| cg10652637 | rs161936 | A | G | -0.021 | 0.002 | 1.75E-17 | 0.324 | -0.288 |  |
| cg10831642 | rs7068070 | G | A | -0.038 | 0.005 | 1.19E-15 | 0.222 | -0.271 | SH3PXD2A |
| cg11938718 | rs10198351 | C | T | -0.033 | 0.003 | 1.92E-28 | 0.424 | -0.368 | HPCAL1 |
| cg15959270 | rs3848075 | T | C | 0.034 | 0.006 | 2.19E-08 | 0.059 | 0.191 | DNAJC3 |
| cg16583315 | rs1953232 | A | C | -0.032 | 0.003 | 8.75E-21 | 0.394 | -0.315 | MAX |
| cg16599817 | rs3107151 | T | G | 0.021 | 0.004 | 3.24E-09 | 0.396 | 0.202 | PRKCZ |
| cg16985652 | rs12757772 | A | G | 0.026 | 0.003 | 3.03E-14 | 0.369 | 0.258 | NR0B2 |
| cg17738841 | rs10184608 | C | T | -0.033 | 0.005 | 2.32E-09 | 0.080 | -0.204 |  |
| cg18735402 | rs11672660 | T | C | -0.012 | 0.001 | 5.61E-21 | 0.199 | -0.316 | GIPR |
| cg18879389 | rs1079380 | G | A | -0.017 | 0.003 | 2.00E-09 | 0.497 | -0.205 | TFF2 |
| cg18931760 | rs10835958 | T | C | 0.017 | 0.003 | 6.07E-11 | 0.429 | 0.223 | EIF3M |
| cg20080878 | rs2980869 | T | C | 0.020 | 0.003 | 1.27E-12 | 0.489 | 0.241 |  |
| cg20929545 | rs4614 | G | A | -0.029 | 0.003 | 4.14E-18 | 0.405 | -0.293 | HMBS |
| cg23358740 | rs7147774 | T | C | 0.017 | 0.001 | 1.24E-57 | 0.066 | 0.513 | C14orf72 |
| cg23722790 | rs11806284 | T | G | 0.039 | 0.006 | 7.55E-10 | 0.049 | 0.210 | SLC35D1 |
| cg23935522 | rs762785 | G | A | -0.025 | 0.003 | 1.14E-16 | 0.410 | -0.280 | ZDHHC17 |
| cg23975840 | rs7960700 | G | A | 0.046 | 0.005 | 3.16E-17 | 0.179 | 0.285 |  |
| cg24188163 | rs10108662 | A | C | -0.032 | 0.003 | 1.64E-27 | 0.344 | -0.362 | IDO1 |

Figure S 8 Forest plots of comparison between MR Egger and IVW methods used in the asthma to DNA methylation of the MR analysis at the top 20 CpGs from the EWAS of asthma at 7.5 years adjusted for basic cells.


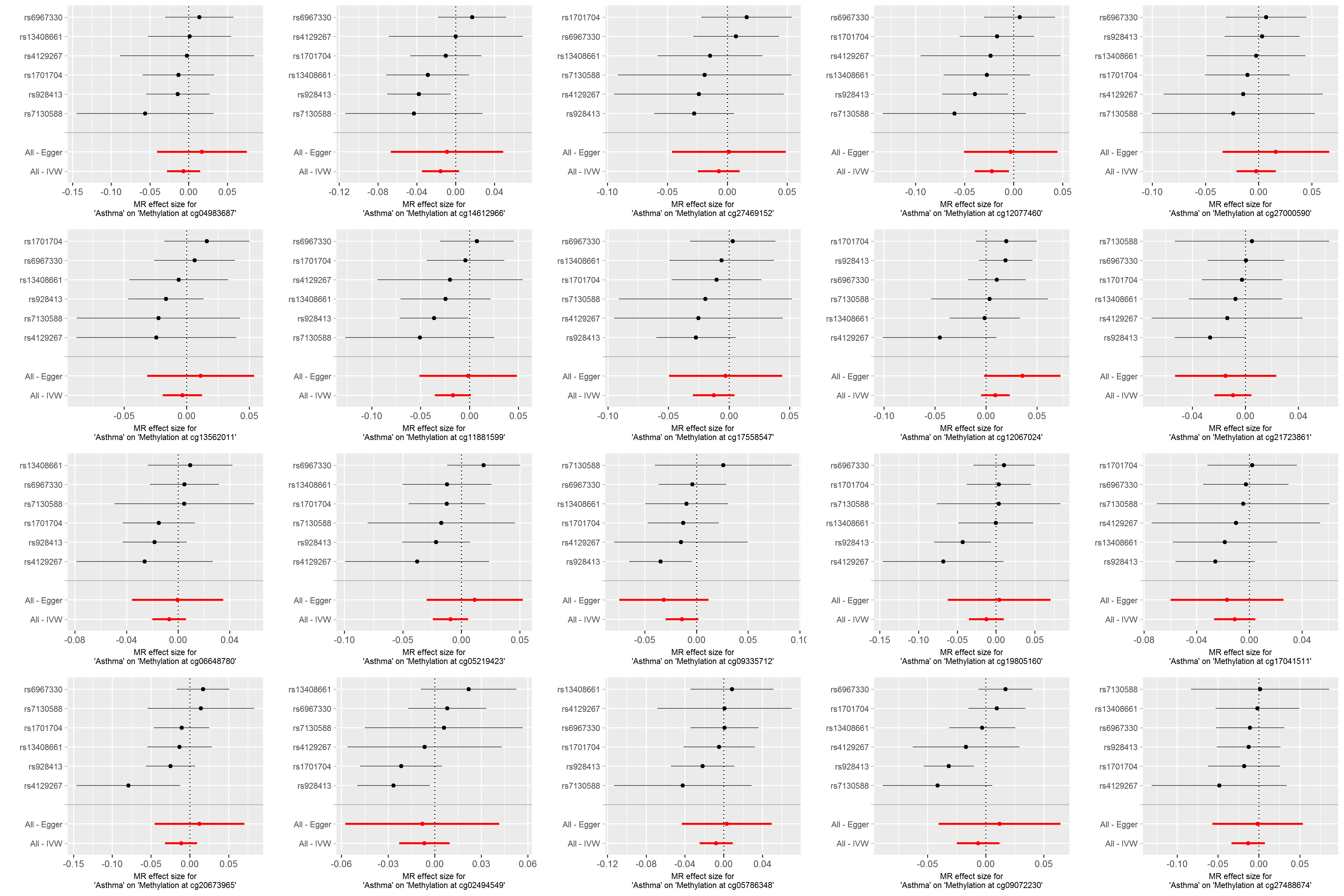


Figure S 9 Scatter plots of comparison between different methods used in the asthma to DNA methylation direction of the MR analysis at the top 20 CpGs.

**
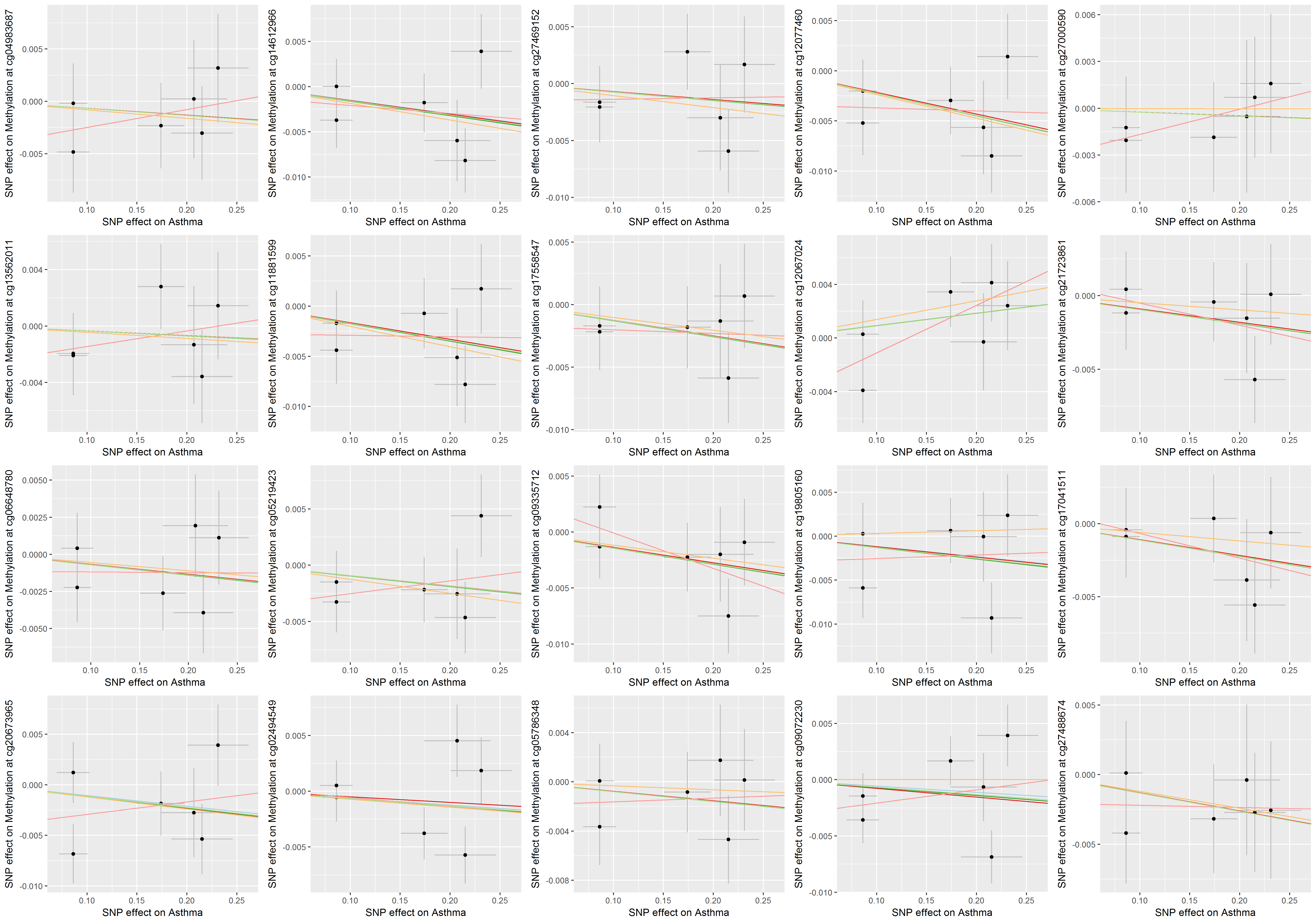
**

Figure S 10 Causal effect of asthma on DNA methylation at the 2 CpGs associated with asthma in the EWAS of current asthma at 16.5 years and methylation at 16.5 years using two-sample MR approach. Obs=Observational association from the EWAS, IVW= Inverse variance-weighted, ML= Maximum Likelihood, WM= Weighted Median, Egger= MR Egger regression.


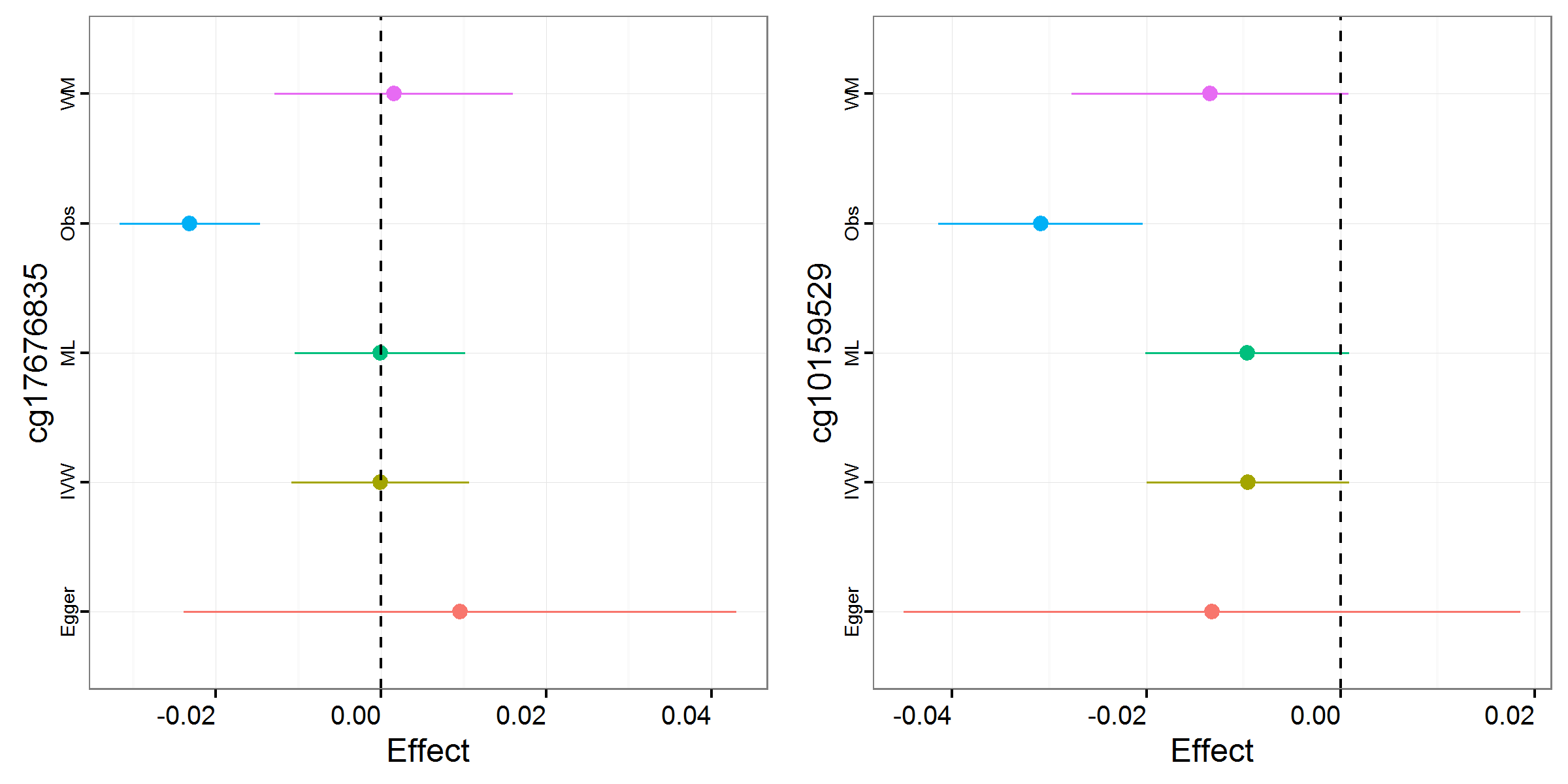


Table S 11 Pairwise comparisons of the 36 hit CpGs reported in a previously published EWAS of IgE levels with the results of EWAS of current asthma at 7.5 years both adjusted and unadjusted for basic cells. Results that replicate in our EWAS (FDR adjusted p-value <0.05) highlighted in red. Note NA for FDR where probes were excluded.

|  |  | **Current asthma at 7.5 years adjusted for basic cells** | | | | **Current asthma at 7.5 years unadjusted** | | | |
| --- | --- | --- | --- | --- | --- | --- | --- | --- | --- |
| **CpG** | **Gene** | **Beta** | **P-value** | **FDR** | **FDR-adj*** | **Beta** | **P-value** | **FDR** | **FDR-adj*** |
| cg00002426 | SLMAP | -0.007 | 0.11003 | 0.9397 | NA | -0.006 | 0.109098 | 0.864453 | NA |
| cg00079056 | SUGT1P1/SPINK4 | -0.014 | 2.24E-06 | 0.005958 | NA | -0.014 | 1.66E-06 | 0.00344 | NA |
| cg01614759 | C10orf25/ZNF22 | -0.032 | 4.78E-06 | 0.009548 | NA | -0.034 | 8.29E-07 | 0.002165 | NA |
| cg01770400 | SERPINC1 | -0.015 | 5.06E-05 | 0.045439 | 0.048578 | -0.016 | 1.78E-05 | 0.018134 | 0.019457 |
| cg01998785 | LPCAT2 | -0.037 | 0.000156 | 0.099542 | NA | -0.038 | 0.0001 | 0.058651 | NA |
| cg02643667 | TFF1 | -0.003 | 0.037826 | 0.852641 | 0.849907 | -0.003 | 0.021361 | 0.688573 | 0.680912 |
| cg03693099 | CEL | -0.019 | 0.000179 | 0.107582 | 0.114305 | -0.020 | 7.47E-05 | 0.048067 | 0.050194 |
| cg05215575 | SEPT12 | -0.014 | 0.09473 | 0.929901 | 0.925088 | -0.017 | 0.051742 | 0.791786 | 0.780949 |
| cg06690548 | SLC7A11 | -0.013 | 0.001515 | 0.359166 | 0.371125 | -0.014 | 0.000746 | 0.207369 | 0.218608 |
| cg08404225 | IL5RA | -0.025 | 0.000348 | 0.157575 | 0.168594 | -0.025 | 0.000272 | 0.1118 | 0.115689 |
| cg09447105 | PDE6H | -0.016 | 0.000413 | 0.175427 | 0.186492 | -0.017 | 0.000123 | 0.06799 | 0.070211 |
| cg09676390 | ADARB1 | -0.021 | 0.100036 | 0.933669 | 0.929737 | -0.021 | 0.08601 | 0.84293 | 0.831853 |
| cg10159529 | IL5RA | -0.025 | 0.00014 | 0.093386 | 0.099599 | -0.025 | 7.21E-05 | 0.0473 | 0.050039 |
| cg11398517 | GTSF1L | -0.023 | 0.000902 | 0.271711 | 0.28419 | -0.025 | 0.000353 | 0.131622 | 0.136234 |
| cg13221796 | RB1 | -0.028 | 0.00274 | 0.459644 | NA | -0.031 | 0.000636 | 0.189448 | NA |
| cg15357945 | PRG2 | -0.026 | 2.02E-05 | 0.026068 | NA | -0.026 | 2.92E-05 | 0.025858 | NA |
| cg15996947 | ATP5S | -0.005 | 0.012506 | 0.719403 | NA | -0.006 | 0.004935 | 0.462108 | NA |
| cg15998761 | MFSD6 | -0.022 | 4.55E-06 | 0.009307 | NA | -0.024 | 9.68E-07 | 0.002437 | NA |
| cg16050349 | PIK3CB | -0.021 | 2.69E-05 | 0.03202 | NA | -0.022 | 9.43E-06 | 0.011765 | NA |
| cg17582777 | EFNA3 | -0.014 | 0.005433 | 0.58442 | 0.5945 | -0.014 | 0.00521 | 0.471503 | 0.471983 |
| cg17749520 | ITGA2B | -0.010 | 0.006407 | 0.614328 | 0.621224 | -0.010 | 0.005724 | 0.487859 | 0.48755 |
| cg17784922 | KEL | -0.014 | 1.09E-05 | 0.016302 | 0.017452 | -0.015 | 2.03E-06 | 0.003942 | 0.004122 |
| cg18254848 | CLC | -0.020 | 0.000242 | 0.127431 | 0.134436 | -0.021 | 9.67E-05 | 0.05729 | 0.05918 |
| cg18783781 | SLC25A33 | -0.046 | 2.81E-06 | 0.006843 | 0.00723 | -0.046 | 1.83E-06 | 0.003666 | 0.003846 |
| cg19881895 | SLC43A3 | -0.005 | 0.112418 | 0.941204 | 0.938032 | -0.006 | 0.050234 | 0.789125 | 0.778795 |
| cg20189937 | ATP5S | -0.015 | 0.002643 | 0.453562 | NA | -0.016 | 0.001314 | 0.276514 | NA |
| cg20503329 | COL15A1 | -0.014 | 6.18E-06 | 0.010902 | NA | -0.014 | 1.99E-06 | 0.003918 | NA |
| cg21627181 | SLC17A4 | -0.019 | 4.00E-05 | 0.039727 | NA | -0.019 | 1.75E-05 | 0.018006 | NA |
| cg21631409 | ALDH3B2 | -0.012 | 0.026313 | 0.8131 | NA | -0.011 | 0.027597 | 0.72103 | NA |
| cg24459209 | PRG3 | -0.014 | 0.003527 | 0.505037 | 0.51851 | -0.015 | 0.001674 | 0.311767 | 0.326862 |
| cg25494227 | C12orf59 | -0.011 | 0.014766 | 0.743837 | 0.745951 | -0.012 | 0.007175 | 0.525159 | 0.521241 |
| cg25636075 | TMEM41A | -0.011 | 0.004725 | 0.558168 | 0.569016 | -0.012 | 0.002667 | 0.374548 | 0.384754 |
| cg26136776 | KLF1 | -0.027 | 1.99E-05 | 0.025832 | 0.027728 | -0.027 | 1.07E-05 | 0.012838 | 0.013747 |
| cg26457013 | TMEM86B | -0.028 | 9.94E-08 | 0.000811 | 0.000861 | -0.029 | 3.10E-08 | 0.000235 | 0.000263 |
| cg26787239 | IL4 | -0.021 | 3.86E-05 | 0.03915 | 0.043174 | -0.021 | 1.52E-05 | 0.016355 | 0.017582 |

*FDR recalculated once our probe exclusions have been applied.

**References**

1. Boyd A, Golding J, Macleod J, Lawlor DA, Fraser A, Henderson J, et al. Cohort Profile: The ‘Children of the 90s’—the index offspring of the Avon Longitudinal Study of Parents and Children. International Journal of Epidemiology. 2012. doi: 10.1093/ije/dys064.

2. Pidsley R, CC YW, Volta M, Lunnon K, Mill J, Schalkwyk LC. A data-driven approach to preprocessing Illumina 450K methylation array data. BMC Genomics. 2013;14:293. Epub 2013/05/02. doi: 10.1186/1471-2164-14-293. PubMed PMID: 23631413; PubMed Central PMCID: PMCPmc3769145.

3. Touleimat N, Tost J. Complete pipeline for Infinium((R)) Human Methylation 450K BeadChip data processing using subset quantile normalization for accurate DNA methylation estimation. Epigenomics. 2012;4(3):325-41. Epub 2012/06/14. doi: 10.2217/epi.12.21. PubMed PMID: 22690668.

4. Naeem H, Wong N, Chatterton Z, Hong MK, Pedersen J, Corcoran N, et al. Reducing the risk of false discovery enabling identification of biologically significant genome-wide methylation status using the HumanMethylation450 array. BMC Genomics. 2014;15(1):51. PubMed PMID: doi:10.1186/1471-2164-15-51.

5. Tukey J. Exploratory Data Analysis. Addison-Wesley. 1977:p43-4.

6. Shim H, Chasman DI, Smith JD, Mora S, Ridker PM, Nickerson DA, et al. A Multivariate Genome-Wide Association Analysis of 10 LDL Subfractions, and Their Response to Statin Treatment, in 1868 Caucasians. PLOS ONE. 2015;10(4):e0120758. doi: 10.1371/journal.pone.0120758.

7. Teslovich TM, Musunuru K, Smith AV, Edmondson AC, Stylianou IM, Koseki M, et al. Biological, clinical and population relevance of 95 loci for blood lipids. Nature. 2010;466(7307):707-13. Epub 2010/08/06. doi: 10.1038/nature09270. PubMed PMID: 20686565; PubMed Central PMCID: PMCPMC3039276.

8. Brion M-JA, Shakhbazov K, Visscher PM. Calculating statistical power in Mendelian randomization studies. International Journal of Epidemiology. 2013;42(5):1497-501. doi: 10.1093/ije/dyt179.

9. Welter D, MacArthur J, Morales J, Burdett T, Hall P, Junkins H, et al. The NHGRI GWAS Catalog, a curated resource of SNP-trait associations. Nucleic Acids Research. 2014;42(D1):D1001-D6. doi: 10.1093/nar/gkt1229.

10. Ramasamy A, Kuokkanen M, Vedantam S, Gajdos ZK, Couto Alves A, Lyon HN, et al. Genome-Wide Association Studies of Asthma in Population-Based Cohorts Confirm Known and Suggested Loci and Identify an Additional Association near HLA. PLoS One. 2012;7(9). doi: 10.1371/journal.pone.0044008. PubMed PMID: 23028483; PubMed Central PMCID: PMCPMC3461045.

11. Hirota T, Takahashi A, Kubo M, Tsunoda T, Tomita K, Doi S, et al. Genome-wide association study identifies three new susceptibility loci for adult asthma in the Japanese population. Nat Genet. 2011;43(9):893-6. Epub 2011/08/02. doi: 10.1038/ng.887. PubMed PMID: 21804548; PubMed Central PMCID: PMCPMC4310726.

12. Ferreira MA, Matheson MC, Duffy DL, Marks GB, Hui J, Le Souef P, et al. Identification of IL6R and chromosome 11q13.5 as risk loci for asthma. Lancet. 2011;378(9795):1006-14. Epub 2011/09/13. doi: 10.1016/s0140-6736(11)60874-x. PubMed PMID: 21907864; PubMed Central PMCID: PMCPMC3517659.

13. Bonnelykke K, Sleiman P, Nielsen K, Kreiner-Moller E, Mercader JM, Belgrave D, et al. A genome-wide association study identifies CDHR3 as a susceptibility locus for early childhood asthma with severe exacerbations. Nat Genet. 2014;46(1):51-5. Epub 2013/11/19. doi: 10.1038/ng.2830. PubMed PMID: 24241537.

14. Gaunt TR, Shihab HA, Hemani G, Min JL, Woodward G, Lyttleton O, et al. Systematic identification of genetic influences on methylation across the human life course. Genome Biology. 2016;17(1):1-14. doi: 10.1186/s13059-016-0926-z.
